# Nebula/DSCR1 Upregulation Delays Neurodegeneration and Protects against APP-Induced Axonal Transport Defects by Restoring Calcineurin and GSK-3β Signaling

**DOI:** 10.1371/journal.pgen.1003792

**Published:** 2013-09-26

**Authors:** Jillian L. Shaw, Karen T. Chang

**Affiliations:** 1 Zilkha Neurogenetic Institute and Department of Cell & Neurobiology, Keck School of Medicine, University of Southern California, Los Angeles, California, United States of America; 2 Neuroscience Graduate Program, University of Southern California, Los Angeles, California, United States of America; Stanford University School of Medicine, United States of America

## Abstract

Post-mortem brains from Down syndrome (DS) and Alzheimer's disease (AD) patients show an upregulation of the Down syndrome critical region 1 protein (DSCR1), but its contribution to AD is not known. To gain insights into the role of DSCR1 in AD, we explored the functional interaction between DSCR1 and the amyloid precursor protein (APP), which is known to cause AD when duplicated or upregulated in DS. We find that the *Drosophila* homolog of DSCR1, Nebula, delays neurodegeneration and ameliorates axonal transport defects caused by APP overexpression. Live-imaging reveals that Nebula facilitates the transport of synaptic proteins and mitochondria affected by APP upregulation. Furthermore, we show that Nebula upregulation protects against axonal transport defects by restoring calcineurin and GSK-3β signaling altered by APP overexpression, thereby preserving cargo-motor interactions. As impaired transport of essential organelles caused by APP perturbation is thought to be an underlying cause of synaptic failure and neurodegeneration in AD, our findings imply that correcting calcineurin and GSK-3β signaling can prevent APP-induced pathologies. Our data further suggest that upregulation of Nebula/DSCR1 is neuroprotective in the presence of APP upregulation and provides evidence for calcineurin inhibition as a novel target for therapeutic intervention in preventing axonal transport impairments associated with AD.

## Author Summary

Alzheimer's disease (AD) is a debilitating neurodegenerative disease characterized by gradual neuronal cell loss and memory decline. Importantly, Down syndrome (DS) individuals over 40 years of age almost always develop neuropathological features of AD, although most do not develop dementia until at least two decades later. These findings suggest that DS and AD may share common genetic causes and that a neuroprotective mechanism may delay neurodegeneration and cognitive decline. It has been shown that the amyloid precursor protein (APP), which is associated with AD when duplicated and upregulated in DS, is a key gene contributing to AD pathologies and axonal transport abnormalities. Here, using fruit fly as a simple model organism, we examined the role of Down syndrome critical region 1 (DSCR1), another gene located on chromosome 21 and upregulated in both DS and AD, in modulating APP phenotypes. We find that upregulation of DSCR1 (Nebula in flies) is neuroprotective in the presence of APP upregulation. We report that *nebula* overexpression delays the onset of neurodegeneration and transport blockage in neuronal cells. Our results further suggest that signaling pathways downstream of DSCR1 may be potential therapeutic targets for AD.

## Introduction

Virtually all Down syndrome (DS) adults develop progressive neurodegeneration as seen in Alzheimer's disease (AD), and overexpression of the amyloid precursor protein (*APP*), a gene located on chromosome 21, is thought to contribute to AD in DS [1]–[3]. Consistently, duplication of a normal copy of *APP* is sufficient to cause familial AD [4], [5], confirming that it is a key gene in AD neuropathologies seen in DS. This well-known connection between AD and DS provides a unique opportunity to identify the genetic and molecular pathways contributing to AD. In addition to *APP*, another gene likely to play a crucial role in both AD and DS is the Down syndrome critical region 1 gene (*DSCR1*, also known as *RCAN1*). Intriguingly, post-mortem brains from AD patients show increased *DSCR1* both at mRNA and protein levels [6]–[8]. Studies have also shown that oxidative stress and Aβ42 exposure can induce *DSCR1* expression [8], [9]. *DSCR1* is located on human chromosome 21 and encodes a highly conserved calcineurin inhibitor family called calcipressin [10]–[15]. DSCR1 has been implicated paradoxically in both promoting cell survival in response to oxidative stress and in inducing apoptosis [8], [9], [16], [17]. The role of DSCR1 in AD thus remains unclear and an important question is whether DSCR1 contributes to AD or plays a role in combating the toxic effects of APP overexpression.

To elucidate the role of DSCR1 in modulating APP-induced phenotypes, we used *Drosophila* as a model system, which has been used successfully to investigate various human neurodegenerative diseases including AD, Parkinson's, and polyglutamine-repeat diseases [18]–[27]. Overexpression of *APP* in both fly and mouse models have previously been shown to cause age-dependent neurodegeneration and axonal transport defects [28]–[31]. Furthermore, impaired transport of essential organelles and synaptic vesicles caused by APP perturbation is thought to be an underlying cause of synaptic failure and neurodegeneration in AD [32]–[34]. However, mechanisms for how APP induces transport defects remain unclear. Here, we show that Nebula, the fly homolog of DSCR1, delays neurodegeneration and reduces axonal transport defects caused by *APP* overexpression. We report that Nebula enhances anterograde and retrograde axonal trafficking as well as the delivery of synaptic proteins to the synaptic terminal. We find that APP upregulation elevates calcineurin activity and GSK-3β signaling, but Nebula co-upregulation corrects altered signaling to restore axonal transport. Together, our results indicate that Nebula/DSCR1 upregulation is neuroprotective in the presence of *APP* overexpression and further suggest that Nebula/DSCR1 upregulation may delay AD progression. In addition, our results for the first time link defective calcineurin signaling to altered axonal transport and imply that restoring calcineurin and GSK-3β signaling may be a feasible strategy for treating AD phenotypes caused by APP upregulation.

## Results

### Upregulation of Nebula Delays APP-Induced Neurodegeneration

**Figure 1:**
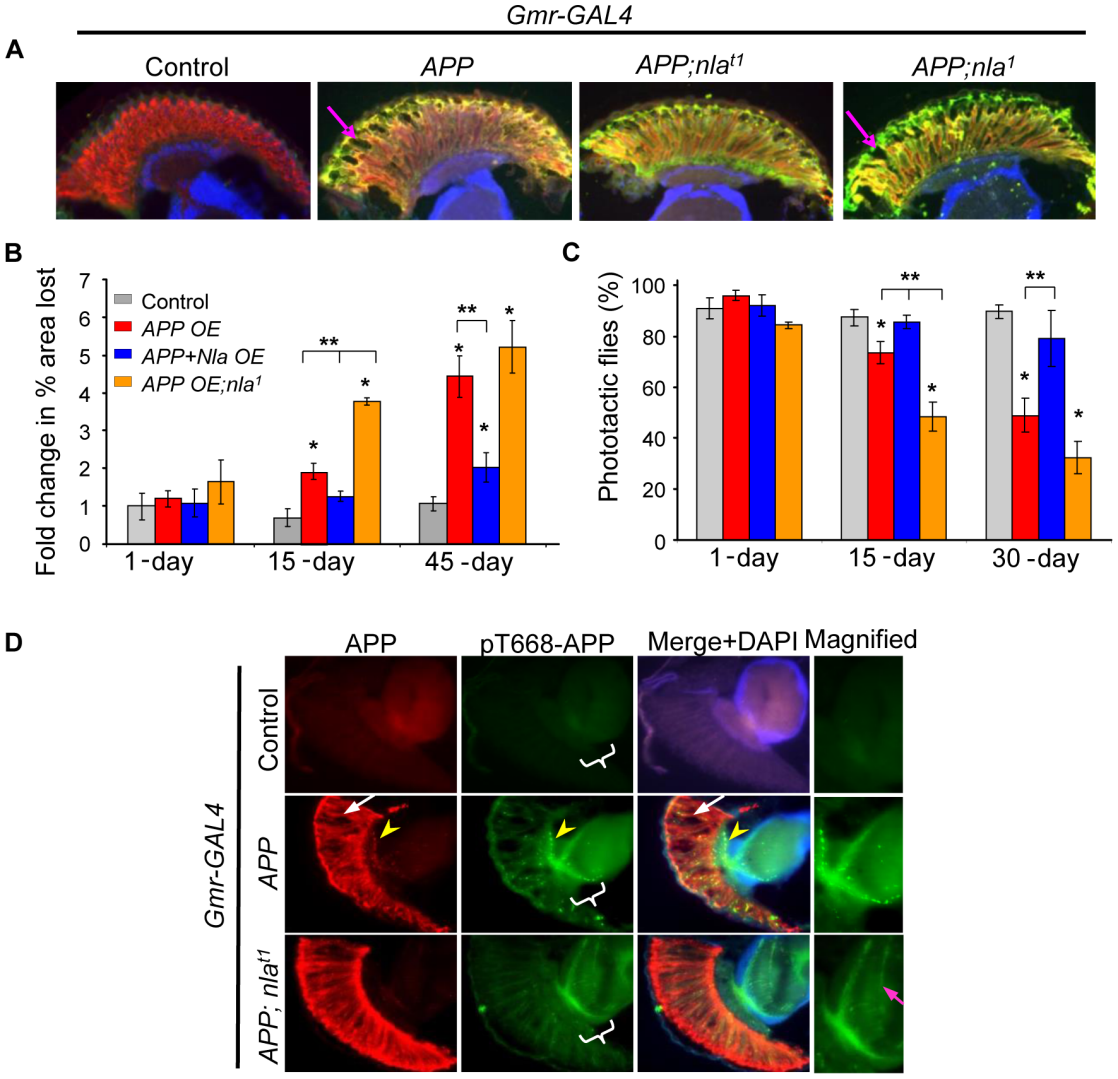
Nebula overexpression reduces APP-induced degeneration structurally and functionally. (A) Cryostat section of 15-day old flies. Neurodegeneration is seen as holes in the fly retina (arrow). Photoreceptor neurons were detected with mAb24B10 (red) and N-APP antibody (green). To normalize the number of transgenes found in different fly lines, control, APP overexpression (OE), or *APP;nla^1^* flies also carry one copy of *UAS-LacZ* gene driven by *Gmr-GAL4*. (B) Fold change in % area lost. n>4 heads per genotype and age. (C) Percentage of flies that moved toward light. n = 3–4 separate tests, total >100 flies per genotype. All values are mean ± S.E.M, * p≤0.05 compared to control, ** P<0.05 compared to the indicated genotypes. (D) Sections of 45 day old fly heads stained with mAb6E10 (APP) and with antibody specific for pT668-APP. White arrow highlights vacuoles and yellow arrowhead points to aggregates. The medulla in which R7-8 terminate is magnified on the right. More pT668-APP is seen in the axon terminals of the photoreceptor neurons (highlighted by magenta arrow).

To examine the role of DSCR1 in modulating APP-induced neurodegeneration and axonal transport defects, we generated transgenic flies containing *UAS-APP* (APP) in the presence or absence of *UAS*-*nebula* (*nla^t1^*) [15]. Targeted expression of human *APP* in the fly eyes using the *Gmr-GAL4* driver caused age-dependent degeneration of the photoreceptor neurons, consistent with a previous report by Greeve et al [35]. As seen in Fig. 1A, staining with an antibody specific for the photoreceptor neurons (24B10) and antibody against the APP protein (6E10) revealed the presence of vacuoles in the retina (arrow). Surprisingly, overexpression of *nebula* together with *APP* (*APP;nla^t1^*) reduced neurodegeneration (as determined by calculating the fold change in the percentage of area lost), suggesting that Nebula upregulation is neuroprotective (Figs. 1A and 1B). By 45-days of age, flies expressing both *nebula* and *APP* started to show increased vacuole formation, but the extent of degeneration was significantly reduced compared to that of *APP* overexpression, further implying that Nebula delays the onset of neurodegeneration rather than completely preventing it.

To confirm that Nebula indeed protects against neurodegeneration caused by APP upregulation, we expressed *APP* in *nla^1^*, a previously characterized *nla* hypomorphic mutant [15]. Note that because *nebula* null alleles are lethal [15], *nebula* hypomorphs were examined. Fig. 1 shows that decreasing Nebula level enhanced APP-induced neurodegeneration in the retina (*APP;nla^1^*), thus highlighting the importance of endogenous Nebula protein in conferring neuroprotection. We did not detect significant neurodegeneration in *nla^1^* mutant and *nla* overexpression flies even by 45 days of age (data not shown), indicating that APP is necessary for the observed phenotype. In addition, mitigation of photoreceptor degeneration by Nebula upregulation is not due to altered expression level of *APP*, since *UAS-LacZ* transgene was included to balance out the number of transgenes (we found *Gmr-GAL4* is particularly sensitive to number of transgenes). The level of APP protein in each fly line is also further confirmed by staining with the 6E10 antibody (Fig. 1A) and Western blot analyses (Fig. S1). Comparable level of APP was detected in all transgenic lines, suggesting that rescue by *nebula* overexpression is not due to altered APP level.

We next determined if Nebula rescues functional defects in photoreceptor by measuring the ability of flies to see light. Flies are normally phototactic and will move toward light when placed in test tubes with light source on the opposite end [36]. We find that the severity of the vacuole phenotype was paralleled by impairments in phototactic behavior (Fig. 1C). Flies overexpressing *APP* showed age-dependent decline in phototaxis that is delayed by *APP* and *nebula* expression (Fig. 1C). Taken together, these results imply that *nebula* overexpression protects neurons structurally as well as functionally against the toxic effects of *APP* overexpression.

### Nebula Upregulation Ameliorates APP-Induced Aggregate Formation in Axons

We also noticed that *APP* overexpression caused formation of APP aggregates in the photoreceptor axons as detected by 6E10 antibody (Fig. 1D; yellow arrow head). Previous studies have shown that APP phosphorylated on threonine 668 (pT668-APP) is preferentially transported in axons [37], we thus further monitored the distribution of pT668-APP. We found that overexpression of *APP* led to pT668-APP accumulations in the photoreceptor axons, whereas *APP* and *nebula* co-overexpression significantly enhanced the delivery of pT668-APP to synaptic terminals in the medulla (Fig. 1D). These results suggest that *APP* overexpression may lead to blocked transport that is alleviated by Nebula.

**Figure 2:**
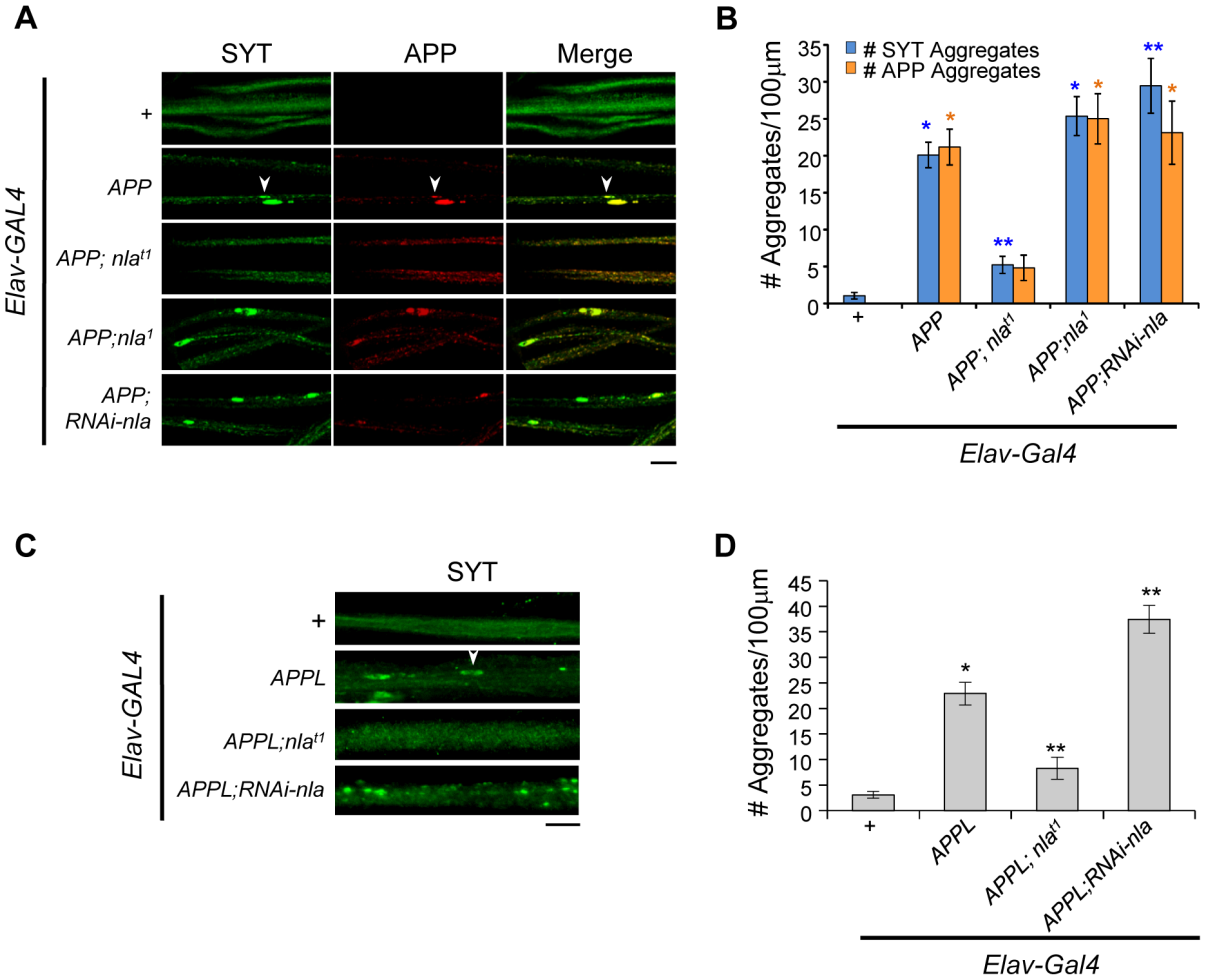
Nebula upregulation rescues APP-dependent aggregate accumulations in axons. (A) Images showing 3^rd^ instar segmental nerves stained with the indicated antibodies. Arrowhead points to an example of aggregate found in axon. APP and synaptotagmin aggregates frequently colocalize. (B) The number of synaptotagmin (SYT) and APP aggregates in axons. n≥10 experiments. * p≤0.05 compared to control, ** P≤0.05 compared to control and *APP* overexpression. (C) Synaptotagmin aggregates in the segmental nerves of 3^rd^ instar larvae with APPL upregulation. (D) Quantification of the number of SYT aggregates. n≥4 independent experiments. All values represent mean ± S.E.M, * p≤0.05 compared to control, ** P≤0.05 compared to control and APPL overexpression. Scale bars = 10 µm.

Axonal transport abnormalities are thought to precede the onset of AD [30], and *APP* overexpression has been shown to cause synaptic vesicle accumulations indicative of blocked axonal transport [28], [29]. We thus further investigated the role of Nebula in modulating APP-induced vesicle aggregation in larval motor axons, which is an excellent system for monitoring vesicle transport because of the long axons and stereotypical innervation of the neuromuscular junction (NMJ). As seen in Fig. 2A, *APP* overexpression in neurons using the *Elav-GAL4* driver caused synaptic vesicle accumulation as detected by synaptotagmin staining in the motor axons, suggesting abnormal vesicle transport. Staining using the 4G8 antibody to detect APP revealed that APP aggregates frequently colocalized with synaptotagmin aggregates, implying that synaptotagmin and APP are either comparably inhibited by physical blockade within the nerve or that they are transported together as suggested by recent reports [38], [39]. Co-upregulation of Nebula and APP significantly prevented APP-induced synaptotagmin and APP accumulations. Decreasing Nebula by crossing it into *nla^1^* background increased the number of synaptotagmin and APP aggregates slightly, although not significantly (Fig. 2B). As *nla^1^* only reduces Nebula level by about 30% and that *nla* null alleles are lethal [15], we used RNAi strategy to further decrease Nebula level (Fig. S2). Figs. 2A–2B show that greater reduction in Nebula level using the *UAS-nla-RNAi* transgene (*RNAi-nla*) further exacerbated the APP-induced aggregation phenotype. To ensure that the observed rescue in phenotype is not due to altered APP overexpression, we monitored the level of neuronal APP protein, as well as Nebula, in different fly lines. As seen in Fig. S3, APP level was unaltered in flies containing different number of transgenes, and Nebula manipulations in *APP* overexpression background showed the expected changes. Similar results were obtained when performing western blot analyses using brains dissected from 3^rd^ instar larvae (Fig. S4). Together, these results confirm that rescue of APP phenotype by Nebula is not due to altered APP expression. In addition, we examined the effect of altering Nebula levels alone on vesicle accumulation. Manipulations of Nebula levels alone did not cause synaptotagmin aggregate accumulation in nerves, suggesting the observed phenotype is APP-dependent (Figs. S5A and S5B).

To verify that the synaptotagmin aggregate accumulation phenotype is not due to a non-specific effect of expressing human *APP*, we also monitored the effect of Nebula on modulating endogenous fly *Appl* gene function. Fig. 2C shows that upregulation of APPL in neurons also caused synaptotagmin accumulation in axons. Nebula co-upregulation significantly reduced the number of synaptotagmin aggregates, whereas Nebula reduction using RNAi significantly exacerbated the phenotype (Figs. 2C and 2D). Together, our results support earlier finding that mammalian APP and Drosophila APPL are functionally conserved [40], and further indicate that APP and APPL-induced axonal transport defects are regulated by Nebula in a similar fashion.

**Figure 3:**
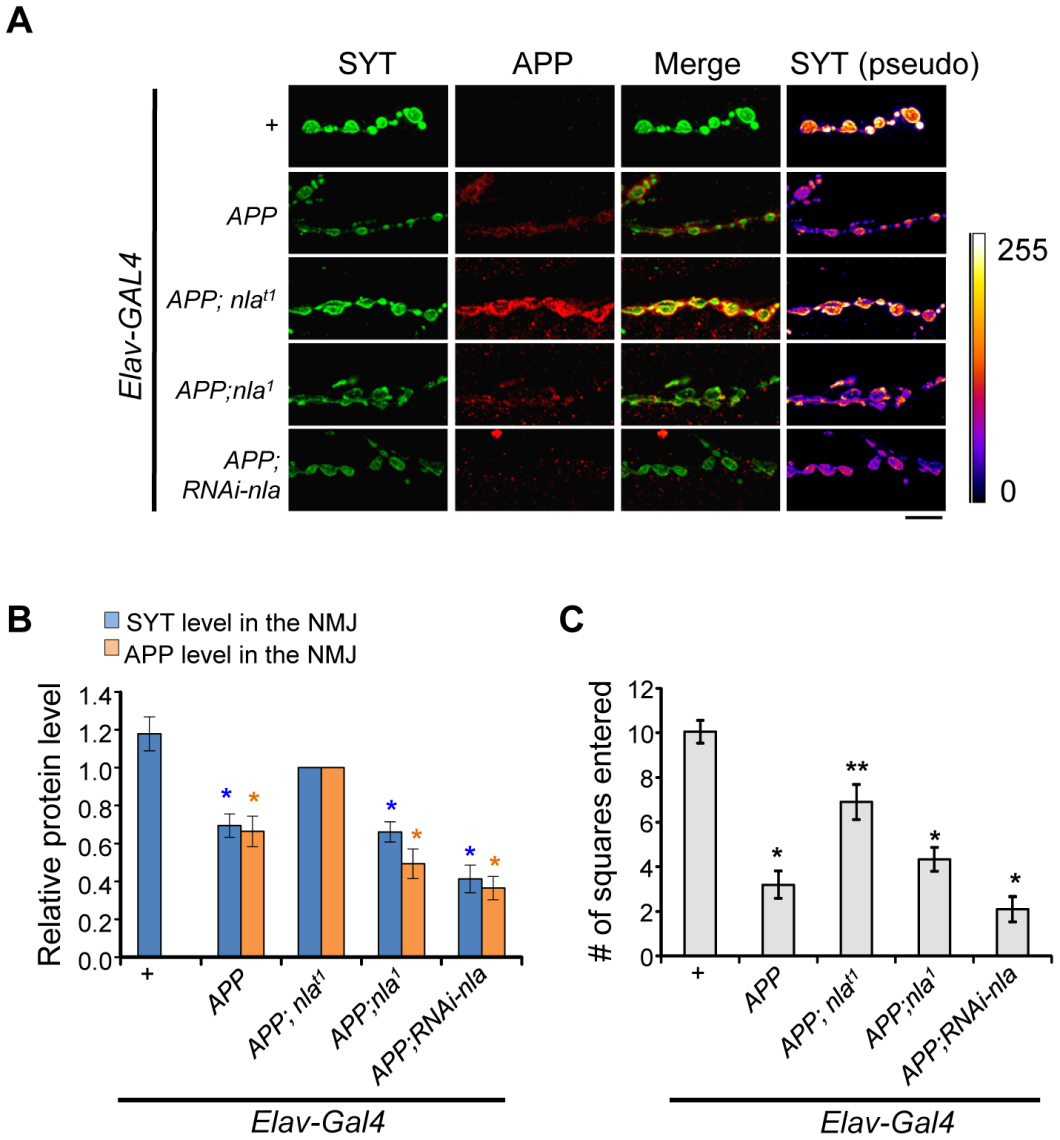
Nebula overexpression restores synaptotagmin level and enhances APP delivery to the synaptic terminals. (A) Representative images of NMJ staining for the indicated genotypes. Scale bars = 10 µm. (B) Levels of SYT and APP in NMJ normalized to flies overexpressing *APP;nla^t1^*. n≥5 independent experiments. (C) Larval locomotor activity assay. n = 10 experiments. All values represent mean ± S.E.M, * p≤0.05 compared to control, ** P<0.05 compared to control and *APP* overexpression.

To determine to what degree aggregate accumulation corresponded to altered delivery of synaptic proteins to the synaptic terminal, we evaluated the levels of both synaptotagmin and APP in the NMJ. As demonstrated in Figs. 3A–3B, APP upregulation significantly reduced the level of average synaptotagmin intensity in the synapse while *nebula* co-overexpression enhanced the delivery of both synaptotagmin and APP to the synaptic terminal. This change is not due to altered overall synaptotagmin or APP levels (Figs. S4 and S5C). Note that the 4G8 antibody does not detect endogenous fly APPL; therefore, we normalized the level of APP delivered to the synapse to flies overexpressing *APP* and *nebula*. We found Nebula reduction did not further reduce the amount of synaptotagmin reaching the terminal (Fig. 3B), albeit it did increase the number of APP-induced aggregates in the axon (Fig. 2B). This result indicates that either retrograde transport of synaptotagmin is altered, or the increase in aggregate number has not yet reached a critical threshold for further impairment. In addition, although no detectable synaptotagmin aggregate was seen in flies with Nebula reduction alone, a decrease in synaptotagmin staining was detected in the synapse (Figs. S5B and S5D). This result suggests that Nebula itself may be required for reliable axonal transport.

We also examined the effect of abnormal aggregate accumulations and reduced delivery of synaptic proteins on locomotor behavior. Overexpression of *APP* dramatically impaired larval movement (Fig. 3C and Movie S1). *Nebula* co-overexpression significantly rescued this locomotor defect, in further support of the hypothesis that Nebula upregulation exerts beneficial effects on synaptic functions by alleviating abnormal aggregate accumulations. Note that further reduction of Nebula in *APP* overexpression background did not significantly worsen the locomotor defect of APP overexpressing larvae, perhaps due to a threshold effect. Reducing Nebula alone was sufficient to induce a mild defect in locomotor activity (Fig. S5E), suggesting delivery of synaptic proteins to the synaptic terminals is crucial for normal synaptic function.

Similar to *APP* overexpression, upregulation of APPL decreased the delivery of synaptotagmin to the synapse. APPL and Nebula co-upregulation showed a higher level of synaptotagmin in the NMJ, confirming Nebula interacts genetically with APPL to rescue impaired in transport (Fig. S6A and S6B). We also found that similar to *RNAi-nla* larvae, *Appl* null mutant (*Appl^d^*) displayed a slight decrease in the level of synaptotagmin at the synapse independent of aggregate accumulation (Figs. S6B and S6C). Reducing Nebula in neurons of *Appl^d^* larvae with the *RNAi-nla* transgene driven by the pan-neuronal *nSyb-GAL4* driver (*Appl^d^; RANi-nla/nSyb-GAL4*) did not further enhance the phenotype, suggesting that the two proteins act in the same pathway to modulate axonal transport.

**Figure 4:**
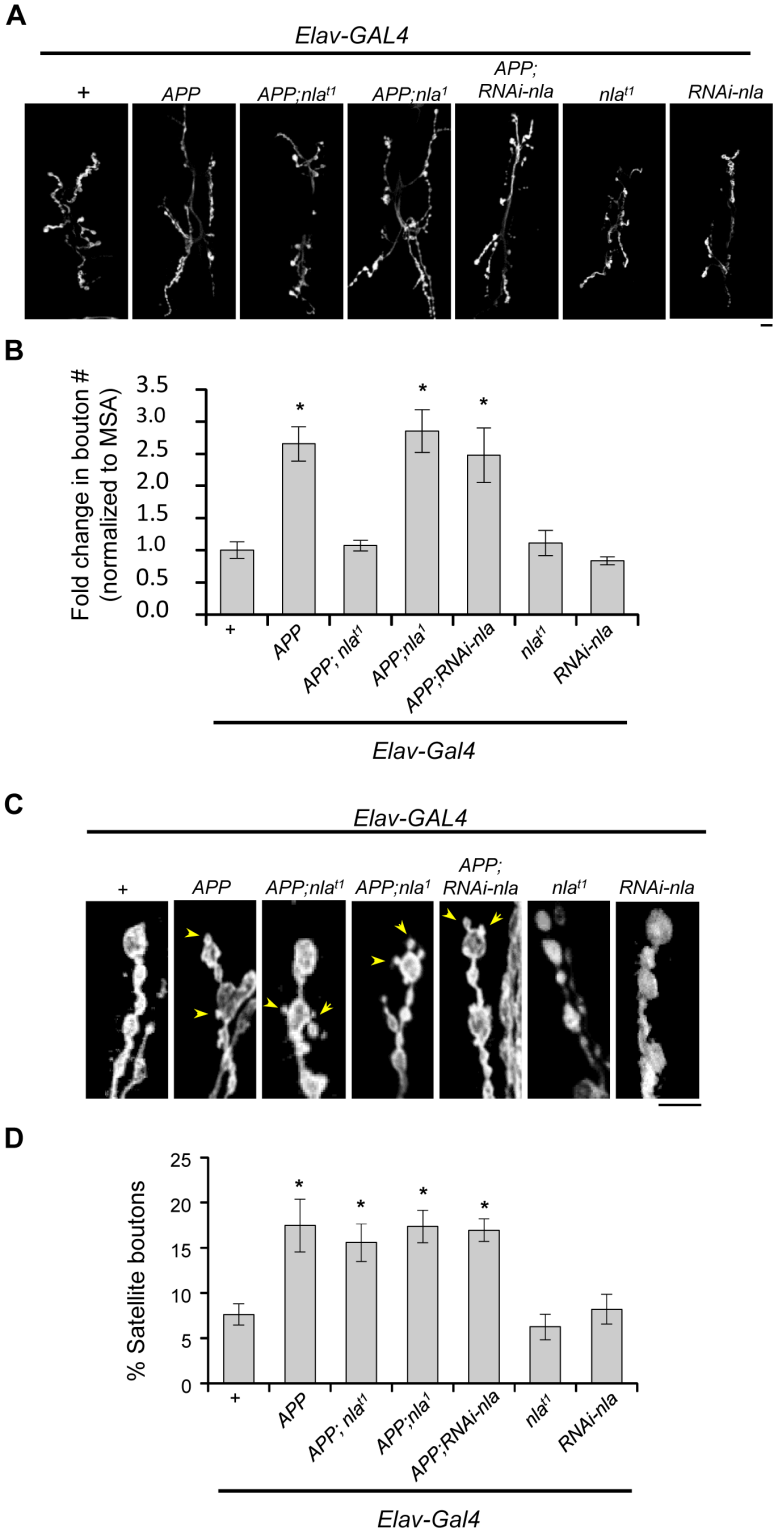
Nebula co-upregulation rescues synaptic bouton over-proliferation caused by APP. (A) Images representing the neuromuscular junction of 3^rd^ instar larvae at segment A2 of muscle segment 6/7. HRP staining outlining synaptic boutons. Scale bar = 10 µm. (B) Quantification of the number of boutons normalized to the muscle surface area (MSA). (C) Magnified images demonstrating the presence of satellite bouton phenotype (arrowheads) in genotypes with APP upregulation. Scale bar = 5 µm. (D) Quantification of the percentage of boutons that are satellite. n≥6 experiments in (B) and (D). All values represent mean ± S.E.M, * p≤0.05 compared to control.

While monitoring synaptotagmin levels at the NMJ, we also noticed that *APP* overexpression triggered changes in synaptic morphology as previously reported [41], [42]. Fig. 4 shows presynaptic terminals stained with HRP to outline the presynaptic terminals, which revealed an increase in the total number of boutons and satellite boutons brought upon by *APP* overexpression. Nebula co-upregulation also rescued APP-induced synapse proliferation phenotype, but not the number of satellite boutons (Fig. 4B and 4D). Manipulating levels of Nebula alone without APP did not influence bouton number or morphology, suggesting that the satellite bouton phenotype is dependent on the presence APP in the synapse. Since reducing Nebula levels alone decreased the delivery of synaptotagmin to the synaptic terminal without altering synaptic morphology, axonal transport problems are not secondary consequences of altered synaptic morphology. A plausible mechanism by which Nebula suppresses the APP-induced over-proliferation phenotype is that Nebula co-upregulation restores the delivery of proteins required for normal synaptic growth such as Fasciclin II (FasII), a cell adhesion molecule shown to influence synaptic morphology [43], [44]. Previous reports suggest that changes in FasII levels differentially affect synaptic growth [42]–[44], and that increasing FasII levels presynaptically can significantly suppress the increase in bouton number observed in APPL overexpression synapses [42]. We therefore quantified FasII levels in the NMJ (Fig. S7). We found that overexpression of *APP* reduced the level of FasII in the NMJ, whereas *APP* and *nebula* co-overexpression restored it (Fig. S7). While APP upregulation may play other roles in synapse formation, these results together with previous reports imply that depletion of FasII in the presynaptic terminal could partially contribute to the hyper-growth phenotype. Furthermore, our data reveal that Nebula upregulation is effective in protecting against multiple phenotypes caused by APP overexpression, including age dependent photoreceptor neurodegeneration, vesicle accumulations in axons, and changes in synaptic morphology.

### Nebula Enhances Anterograde Transport of Amyloid Precursor Protein

**Figure 5:**
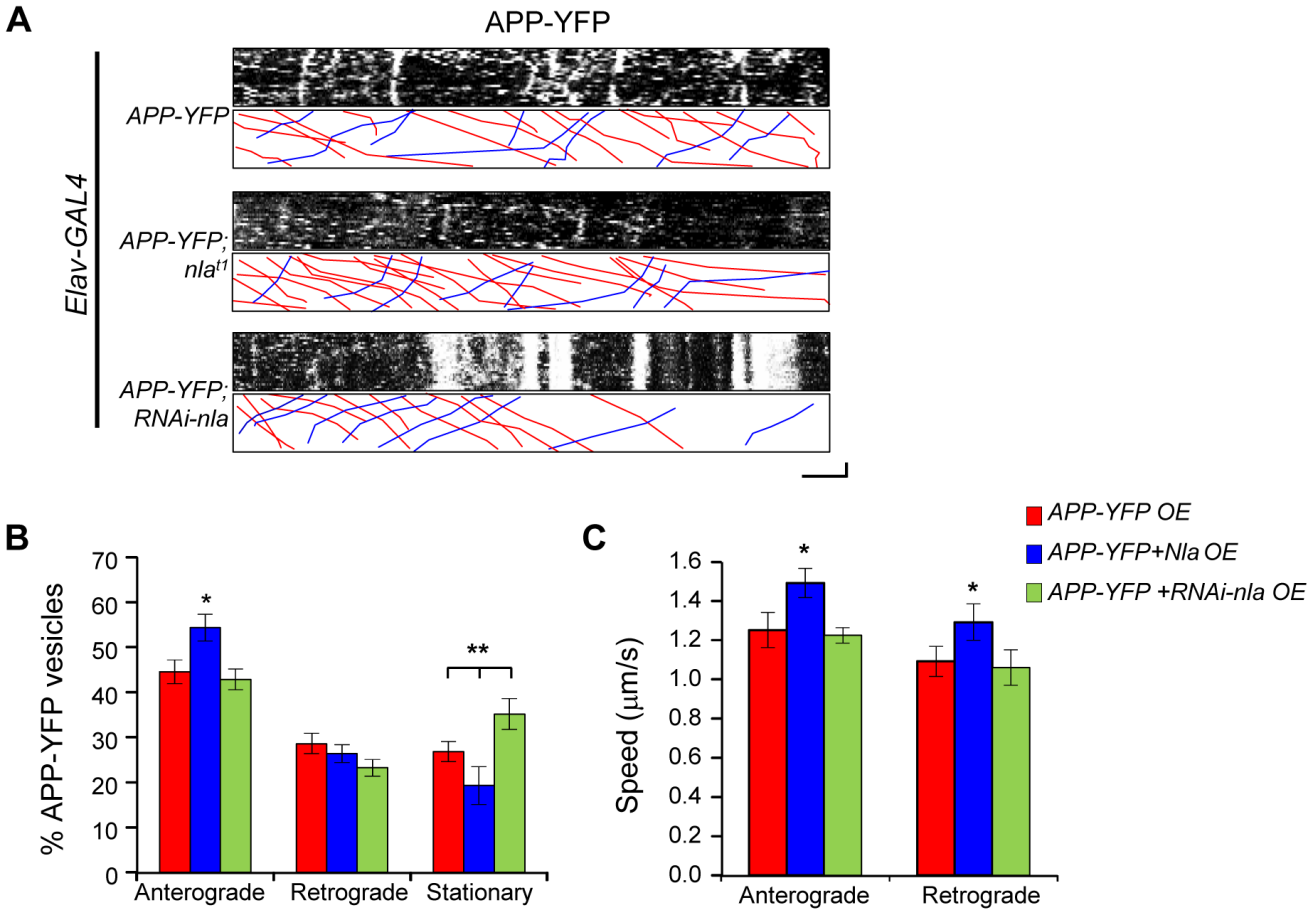
Nebula upregulation enhances anterograde transport of APP-YFP in larval motor axons. (A) Representative kymographs depicting trafficking of APP-YFP vesicles. Stationary vesicles are seen as vertical lines and anterograde movement is depicted as diagonal lines moving from left to right. Scale bars: 5 µm (X) and 30 seconds (Y). Red lines in the lower boxed region highlight anterograde moving vesicles. Blue lines depict retrograde moving vesicles. (B) Quantification of the percentage of anterograde and retrograde moving vesicles, as well as stationary vesicles in the indicated genotypes. *nebula* overexpression significantly increased the relative number of anterograde moving vesicles. (C) Speed of APP-YFP vesicles in the anterograde and retrograde directions. Nebula upregulation enhanced the speed in the anterograde and retrograde directions. All values represent mean ± S.E.M, * p≤0.05 compared to control, ** P<0.05 compared to the indicated genotypes. n≥6 independent experiments per genotype.

To directly evaluate the effect of Nebula on APP transport and to determine whether the observed axonal aggregates correspond to defective axonal transport, we performed live-imaging of human APP tagged with yellow fluorescent protein (APP-YFP). APP-YFP vesicles in larval motor axons displayed movement in both the anterograde and retrograde directions over the 2-minute imaging period as represented by kymographs depicting distance traveled and time in the x- and y-directions, respectively (Fig. 5A). Nebula co-overexpression had a mild, but significant, effect on APP-YFP movement. Nebula co-upregulation increased the percentage of anterograde moving vesicles and resulted in reduced number of stationary APP-YFP; knockdown of Nebula using RNAi increased the number of stationary APP-YFP (Figs. 5A and 5B). Quantification of the average speed of APP-YFP movement revealed that overexpression of *nebula* also increased the speed of APP-YFP movement in both the anterograde and retrograde directions (Fig. 5C). Together, these results suggest that Nebula upregulation enhances the transport of APP, consistent with the decreased aggregate accumulations of APP in axons and increased APP staining in the NMJ when Nebula is co-expressed (Figs. 2A and 3A).

### Nebula Restores Anterograde and Retrograde Trafficking of Synaptic Vesicles and Mitochondria

**Figure 6:**
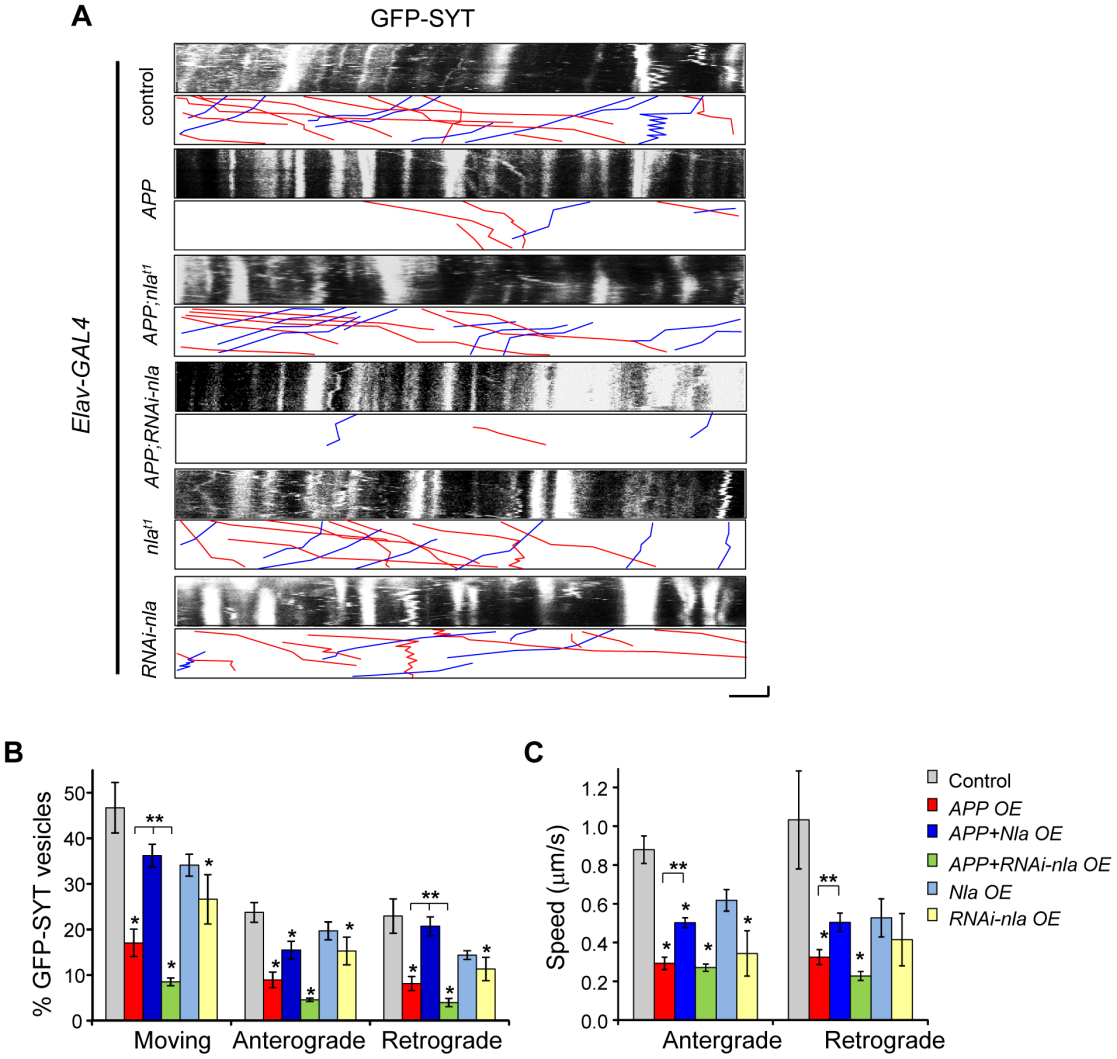
APP upregulation causes defective transport of synaptotagmin that is restored by Nebula co-upregulation. (A) Kymographs depicting movement of GFP-SYT. Scale bars: 5 µm (X) and 60 s (Y). Red lines: anterograde movement; blue lines: retrograde movement. (B) Quantification of the total number of moving vesicles, as well as relative movement in the anterograde and retrograde directions normalized to the total number of observed vesicles. APP overexpression (OE) caused significant defects in the movement of GFP-SYT. (C) Speed of GFP-SYT movement in the anterograde and retrograde directions. Values represent mean ± S.E.M, * p≤0.05 compared to control, ** P<0.05 compared to the indicated genotypes. n≥6 independent experiments per genotype.

To further confirm that Nebula facilitates synaptic vesicle movement in the presence of APP and to better assess the role of endogenous Nebula in regulating transport, we also monitored synaptotagmin movement in the motor axons of larvae expressing GFP-tagged synaptotagmin (GFP-SYT). We find the movement of GFP-SYT to be highly dynamic with anterograde, retrograde, and bi-directional movement (Fig. 6A). Overexpression of *APP* dramatically reduced the percentage of vesicles moving in both the anterograde and retrograde directions while *nebula* co-overexpression significantly facilitated synaptotagmin transport in both directions (Figs. 6A and 6B), albeit retrograde transport was more effectively restored by Nebula. Reducing Nebula using RNAi further diminished APP-induced synaptotagmin transport in both directions, confirming interaction between Nebula and APP. Reduction in the overall movement was also accompanied by a decrease in anterograde and retrograde velocity (Fig. 6C). Together, these results suggest that APP overexpression slows down the overall movement of vesicles, which may lead to accumulation of transported proteins. Nebula co-overexpression with APP partially restores the defect by increasing the movement and speed of transport in both the anterograde and retrograde directions.

To understand the role of endogenous Nebula in axonal transport, we examined the effect of Nebula manipulations on GFP-SYT movement in the absence of *APP* overexpression. We find that Nebula upregulation alone did not significantly influence transport; decreasing Nebula through RNAi was sufficient to reduce the number of moving synaptotagmin vesicle in both directions, as well as the speed of anterograde transport (Fig. 6). This result is consistent with the decrease in synaptotagmin staining in the NMJ seen in static images, and further confirms that Nebula is required for efficient transport of synaptic proteins.

**Figure 7:**
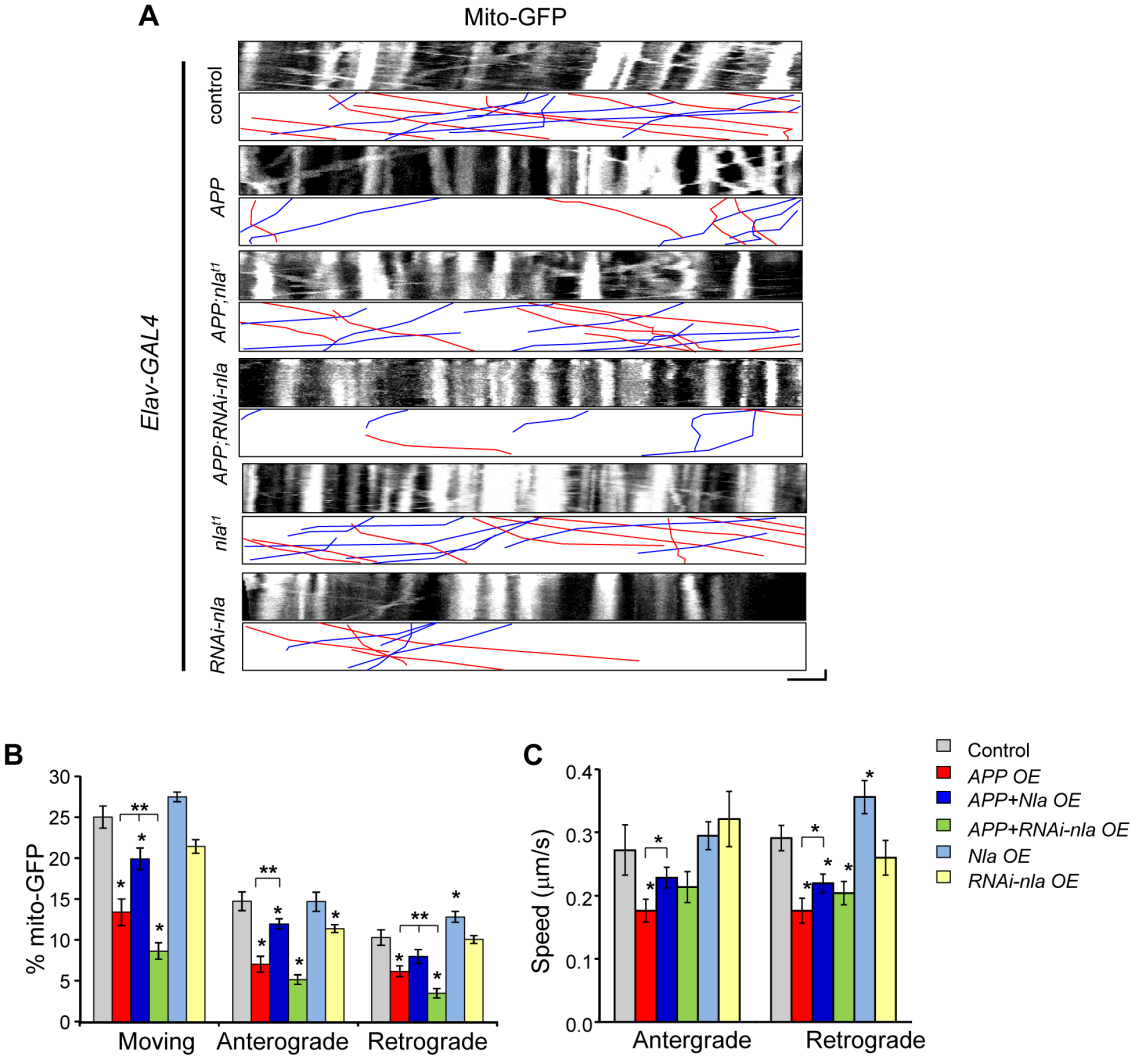
Nebula alters APP-induced mitochondrial transport defects. (A) Kymographs showing mito-GFP movement in motor axons. Scale bars: 5 µm (X) and 60 s (Y). Red lines: anterograde movement; blue lines: retrograde movement. (B) Quantification of the percent of mobile mitochondria, and mitochondria moving in the anterograde and retrograde directions. (C) Speed of mitochondrial movement. All values represent mean ± S.E.M, * p≤0.05 compared to control, ** P<0.05 compared to the indicated genotypes. n≥6 independent experiments per genotype.

To further determine if general axonal transport is affected by APP and Nebula upregulation, we also monitored mitochondrial transport. Proper distribution of mitochondria is vital for normal cell functions and defects in mitochondrial transport can adversely affect cell survival [45]–[47]. Time-lapse live imaging was performed in larvae with GFP targeted to mitochondria (mito-GFP) for the indicated genotypes (Fig. 7). APP upregulation severely impaired the movement of mitochondria in both the anterograde and retrograde directions both in terms of percent in motion and the speed of movement (Figs. 7B and 7C). Nevertheless, the APP-induced mitochondrial transport defect was partially restored by Nebula co-upregulation (Fig. 7 and Movie S2), similar to what was observed for synaptic vesicle transport. Manipulations in the level of Nebula did not significantly alter the overall mitochondrial movement, except that *nebula* overexpression alone seemed to enhance both the proportion and the speed of mitochondria transported in the retrograde direction. This result is consistent with our observation that Nebula co-upregulation was more effective in restoring retrograde GFP-SYT transport. Together, our results suggest that Nebula influences general axonal transport that extends beyond synaptic proteins.

Mitochondria are dynamic organelles whose distribution is tightly regulated to meet the energy demands within the polarized neuron [45], [48]. We find that despite the decrease in mitochondrial movement in flies overexpressing *APP*, the distribution and density of mitochondria within the proximal axon where imaging was performed did not vary across genotypes (Fig. S8A). These results imply that impaired synaptic vesicle transport is not likely caused by local depletion of mitochondria within the axon. Furthermore, mitochondria did not accumulate near the site of synaptotagmin aggregate formation in the axons (Fig. S8B), suggesting that mitochondria are either able to move past the stalled synaptic vesicle accumulations or that mitochondria travel on other non-blocked microtubule tracks.

### *APP* Overexpression Does Not Alter Microtubule Integrity

**Figure 8:**
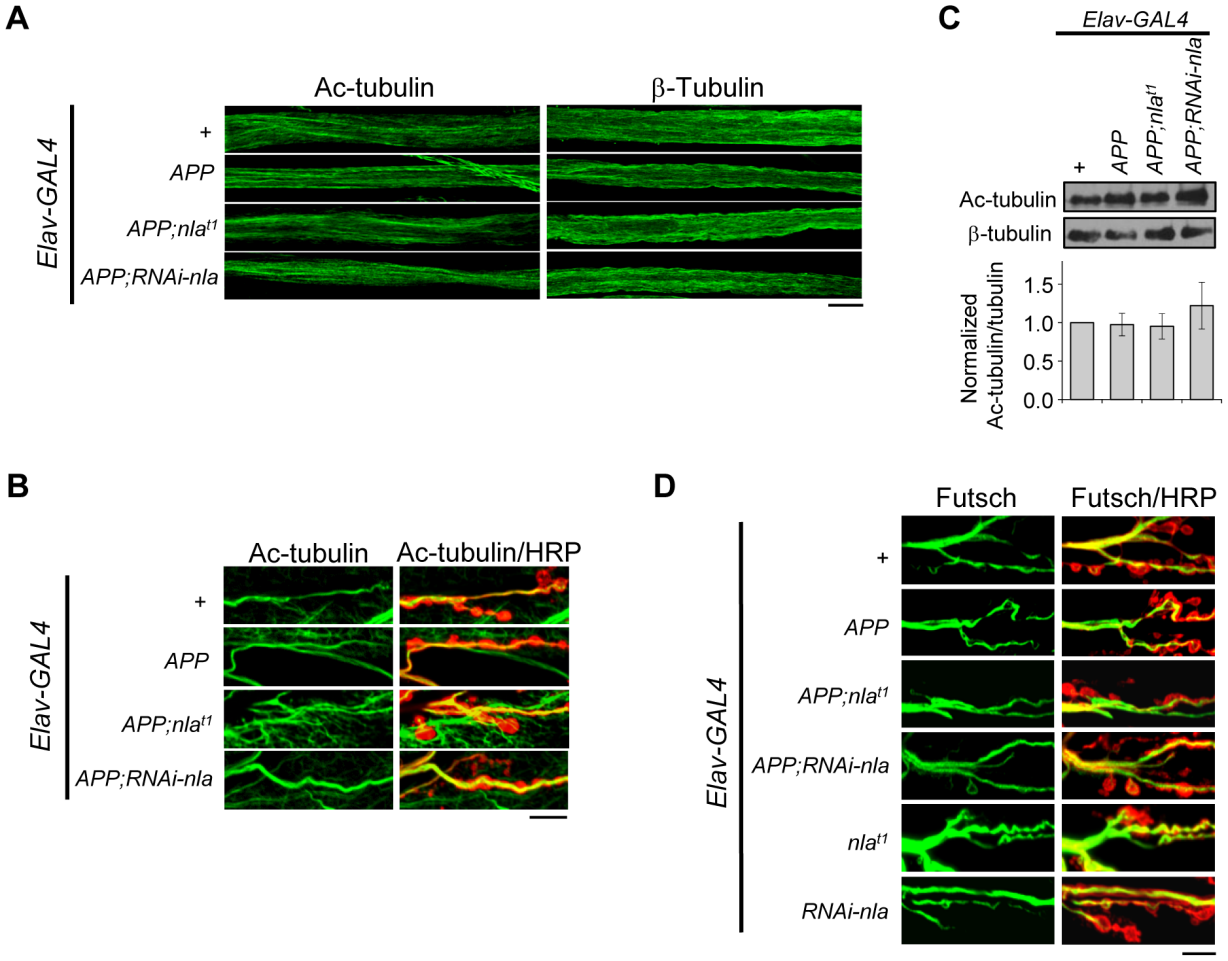
*APP* overexpression does not alter gross microtubule structure in the axons or NMJ. (A) Images of the larval segmental motor axons stained with acetylated tubulin (Ac-tubulin) or β-tubulin as indicated. Microtubule structural integrity was not influenced by *APP* overexpression. (B) Representative images of the larval neuromuscular junction at segment A2 of muscle 6/7 stained with Ac-tubulin (green) and HRP (red). Background Ac-tubulin signal is also detected in the muscle (outside the boundaries of HRP staining). (C) Western blot depicting Ac-tubulin levels for the indicated genotypes. Values were normalized to β-tubulin, which is used as loading control. Lower panel shows quantification of the relative protein level for the indicated transgene normalized to the control. Values represent mean ± SEM, n = 3 independent experiments. (D) Images of the 3^rd^ instar larval NMJ at segment A2 of muscle 6/7 stained with Futsch (green) and HRP (red). All scale bars = 10 µm.

Despite increasing evidence linking defective trafficking of presynaptic proteins, mitochondria, and signaling molecules to neuropathologies of AD, mechanisms for how *APP* overexpression affects axonal transport remain unclear. We first tested the possibility that APP upregulation impairs axonal transport by influencing overall microtubule integrity. To this end, we stained the axonal nerves and NMJs with antibodies against acetylated tubulin, β-tubulin, and Futsch (Fig. 8). Acetylated tubulin is a marker for stable microtubules [49]; Futsch is a microtubule binding protein homolog to human MAP1B and is involved in maintaining microtubule integrity at presynaptic terminals during NMJ growth [50]. Our data revealed that *APP* overexpression did not cause fragmentation of microtubules as revealed by both acetylated tubulin and β-tubulin staining in the axons (Fig. 8A), and filamentous acetylated tubulin staining in the synaptic terminals across all genotypes (Fig. 8B). Note that in Fig. 8B, we also highlighted the presynaptic boutons by HRP staining (red), since acetylated tubulin in the muscles are also detected in the background. Western blot analyses of dissected larval brains further confirmed that the overall level of acetylated tubulin is not altered by *APP* overexpression (Fig. 8C). Closer examination of Futsch staining also did not reveal differences in overall microtubule integrity (Fig. 8D). Together, these results suggest that *APP* overexpression does not cause axonal transport problems by influencing microtubule stability, which is consistent with a recent report that showed normal microtubule stability and acetylated tubulin level in larvae overexpressing *APP-YFP* [51].

### Nebula Mitigates APP-Induced Phenotypes by Regulating Calcineurin and GSK-3β

**Figure 9:**
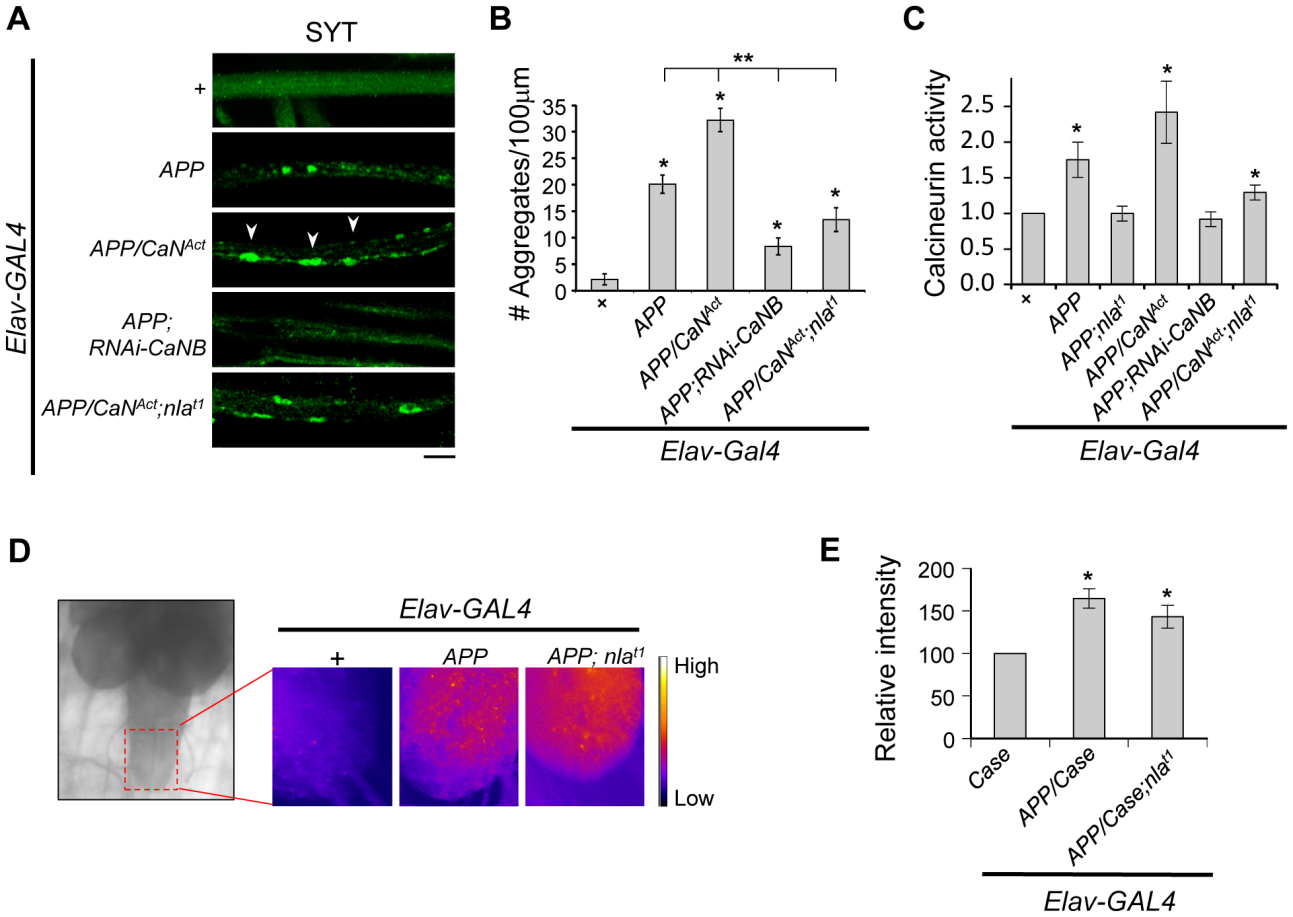
Nebula modulates APP-dependent phenotypes by restoring calcineurin signaling. (A) Representative images of 3^rd^ instar larval motor axons stained with synaptotagmin. Arrowheads point to examples of aggregates. Scale bar: 10 µm. (B) Quantification of the number of aggregates found in each genotype. (C) Calcineurin activity assay. n≥10 independent experiments per genotype for all experiments. (D) Image of 3^rd^ instar brain highlighting the imaging area (left). Magnified images (right) depict the intensity of the genetically encoded fluorescent calcium sensor, Case12, across genotypes. (E) Quantification of the relative fluorescent intensity across genotypes. n = 6 experiments. All values represent mean ± S.E.M. * p≤0.05 compared to control and ** P<0.05 compared to the indicated genotypes.

Nebula encodes an inhibitor of calcineurin that is highly conserved across species [15], we therefore tested the hypothesis that calcineurin inhibition is an underlying mechanism for Nebula-mediated rescue of APP phenotypes. To this end, we genetically altered calcineurin activity in neurons using the UAS/GAL4 strategy. To elevate calcineurin activity, we expressed a constitutively active calcineurin (*CaN^Act^*) with its auto-inhibitory domain deleted (Figs. S9A and S9B). To reduce calcineurin activity, RNAi strategy against the calcineurin B gene (*RNAi-CaNB*), an obligatory subunit necessary for calcineurin activity, was used. We find that similar to Nebula upregulation, decreasing calcineurin using *RNAi-CaNB* in the presence of APP significantly reduced synaptotagmin aggregate accumulations and synaptic depletion, as well as restored larval locomotor behavior (Figs. S9C–E). Overexpression of *CaN^Act^* together with *APP* further exacerbated the APP-induced phenotypes (Figs. 9A and 9B), whereas co-overexpression of *CaN^Act^* and *nebula* diminished the ability of Nebula to protect against APP-induced transport defects. Similar to larvae with reduced levels of Nebula (*RNAi-nla*), larvae expressing *CaN^Act^* did not show aggregate accumulations in axons but displayed a reduced level of synaptotagmin staining in the synapse (Figs. S9D), indicating active calcineurin overexpression alone only has modest effect on axonal transport. As shown above, synaptotagmin aggregate accumulation in nerves and depletion in the synaptic terminals are reliable indicators of significant transport deficiencies; our results thus indicate that Nebula protects against APP-induced defects through inhibition of calcineurin. Furthermore, our data present for the first time that APP upregulation influences axonal transport through activation of calcineurin. This conclusion is further supported by direct measurement of calcineurin activity, in which we find that APP upregulation significantly elevated calcineurin activity but is further restored close to normal in flies overexpressing *APP* and *nebula*, or *APP* and *RNAi-CaNB* (Fig. 9C). Overexpression of *APP*, *CaN^Ac^* ^t^, and *nebula* together showed an intermediate phenotype in both calcineurin activity and aggregate accumulations, suggesting that the severity of aggregate accumulation correlated with the level of calcineurin when APP is upregulated.

How does APP upregulation trigger calcineurin activation? Because calcineurin phosphatase activity is dependent on intracellular calcium concentration [52], we examined the possibility that APP overexpression elevates calcium levels. Using a genetically encoded fluorescent calcium sensor (Case12) previously shown to detect calcium with high sensitivity [53], [54], we compared Case12 signal across different genotypes. Fig. S10 shows that larval brain expressing Case12 displayed a significant increase in signal following application of calcimycin, a calcium ionophore, confirming that the Case12 construct can indeed detect increases in calcium. Overexpression of *APP* alone or overexpression of *APP* and *nebula* also caused a significant elevation in Case12 signal in the larval brain and the ventral ganglion (where the motor neuron cell bodies are located) as compared to the control (Figs. 9D and 9E). These data imply that an APP-mediated increase in calcium is triggering the increase in calcineurin activity. Furthermore, observations that co-overexpression of *APP* and *nebula* increased calcium while simultaneously restoring calcineurin activity indicate that Nebula is influencing axonal transport through calcineurin inhibition rather than acting at a step modulating calcium influx.

**Figure 10:**
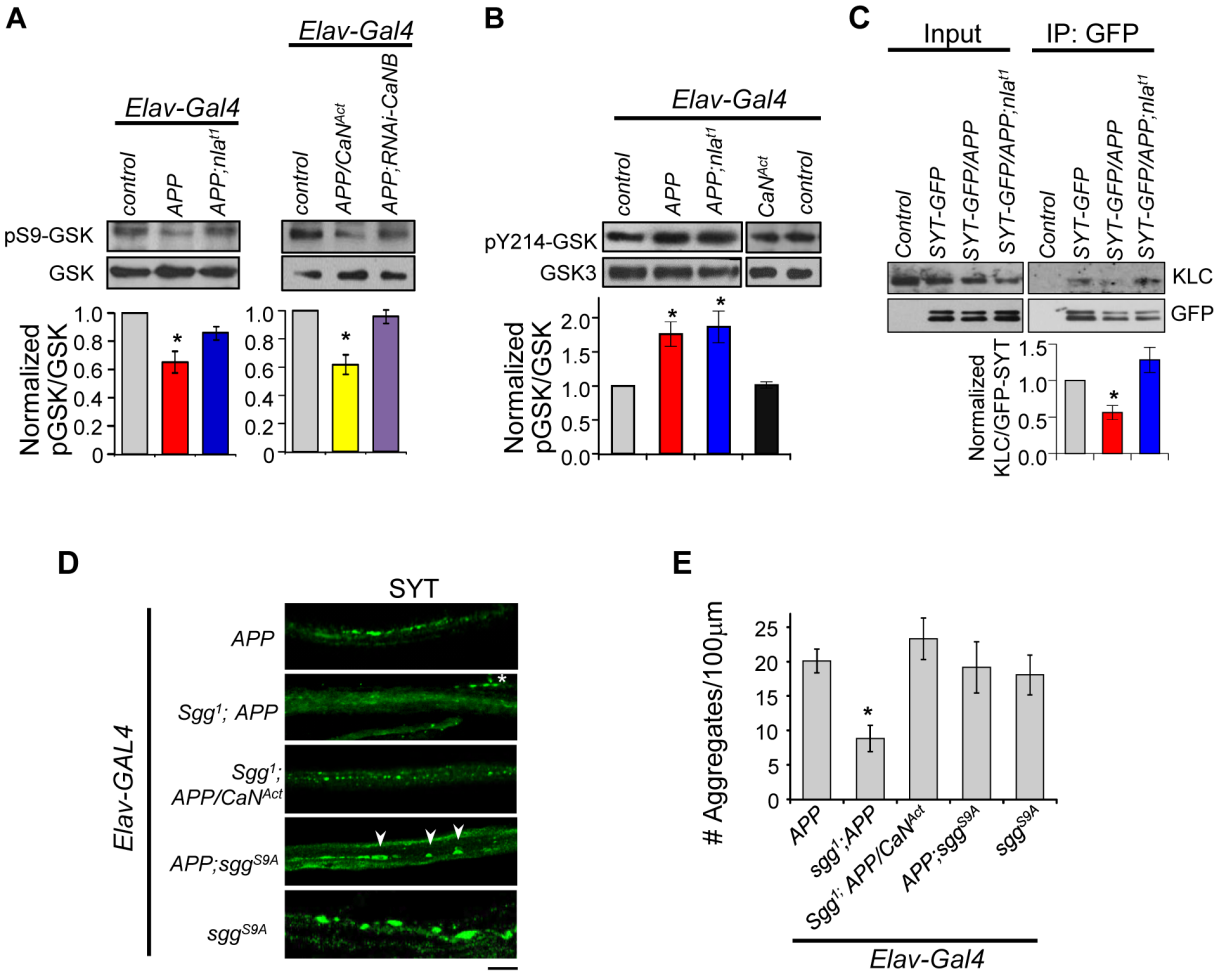
APP upregulation triggers changes in GSK-3β signaling downstream of calcineurin and affects synaptotagmin-kinesin interaction. (A) Western blots performed using antibody specific for GSK-3β phosphorylated on Ser9 (pS9-GSK) and total GSK-3β. (B) Western blot using antibody specific for GSK phosphorylated at Tyr214 in *Drosophila* (pY214-GSK). For (A) and (B) quantification of the ratio of phosphorylated GSK and GSK-3β was normalized to control. n≥3 for each, * p<0.05. (C) Western blots showing reduced interaction between SYT-GFP and kinesin light chain (KLC). Control indicates parallel immunoprecipitation performed using flies that do not express SYT-GFP. n = 3, * p<0.05. (D) Images of 3^rd^ instar larval motor axon stained with synaptotagmin. Arrowheads highlight aggregates and * indicates background staining coming from synaptotagmin in the NMJ. Scale bar: 10 µm. (E) Quantification of the number of aggregates found in each genotype. All values are mean ± S.E.M, * p≤0.05 compared to *APP* overexpression. n≥4 independent experiments per genotype.

Mechanisms by which calcineurin regulates axonal transport are not well understood, but one potential pathway is through regulation of GSK-3β activity. Aberrant activation of GSK-3β has been associated with AD and calcineurin has been shown to activate GSK-3β through dephosphorylation of Ser9 of GSK-3β *in vitro* [55]–[58]. It was suggested that GSK-3β may negatively influence axonal transport by altering microtubule stability through hyperphosphorylation of tau, by inhibiting kinesin motor binding to the cargo through phosphorylation of the kinesin light chain (KLC), or by altering the kinesin motor activity [51], [59]–[61]. These previous findings led us to investigate the possibility that Nebula restores APP-dependent transport problems through calcineurin-mediated regulation of GSK-3β *in vivo*. The activity of GSK-3β is regulated by phosphorylation and dephosphorylation: dephosphorylation of Ser9 by a number of phosphatases including calcineurin is required to activate GSK-3β [56], [62], and phosphorylation at Tyr216 site is necessary to enhance GSK-3β activity [63], [64]. Interestingly, phosphorylation of GSK-3β at Ser9 can both inhibit GSK-3β activity and override the increase in activity even when phosphorylated at Tyr216 [65]. Because these phosphorylation sites are conserved between fly and human, we took advantage of phospho-specific antibodies to monitor GSK-3β activity. Western blot analyses using an antibody specific for phosphorylated Ser9 (pSer9) of GSK-3β revealed that APP upregulation indeed reduced the level of pSer9-GSK-3β while APP and Nebula co-upregulation partially restored the level to normal (Fig. 10A). This suggests APP upregulation leads to GSK-3β activation that is inhibited by Nebula upregulation.

To verify that GSK-3β activation is due to calcineurin activation, we reduced calcineurin activity in *APP* overexpressing flies using *RNAi-CaNB* (Fig. 10A). We find that *APP* and *RNAi-CaNB* co-overexpression in neurons, which was sufficient to restore calcineurin activity, completely prevented GSK-3β dephosphorylation at Ser9 site. This result indicates that APP-induced GSK-3β dephosphorylation at Ser9 is dependent on calcineurin activation *in vivo*. Note that we did not detect enhanced GSK-3β dephosphorylation when *APP* is expressed together with constitutively active calcineurin (*CaN^Act^*), suggesting that calcineurin may in part directly influence transport through GSK-3β-independent pathways.

Our data strongly implicate activation of calcineurin and subsequent GSK-3β induction to be a mechanism underlying APP-induced aggregate phenotype. Because activation of calcineurin alone did not result in synaptotagmin aggregate accumulation, we further hypothesized that APP upregulation also enhances GSK-3β activity through phosphorylation at Tyr216. Western blot analyses show that the level of phosphorylated GSK-3β at Tyr214 (conserved Tyr216 site in *Drosophila*) is indeed elevated in flies overexpressing *APP* or *APP* and *nebula* (Fig. 10B). Overexpression of *CaN^Act^* alone, however, failed to induce phosphorylation at Tyr214, suggesting that phosphorylation of Tyr214 is not affected by calcineurin and dependent on the presence of APP. Together, our data demonstrate that in addition to activating GSK-3β by relieving inhibition through calcineurin, APP upregulation further enhances GSK-3β activity through phosphorylation at Y214 in fly.

Active GSK-3β had been shown to phosphorylate KLC, leading to detachment of the cargo from the motor [59], [66]. Since synaptotagmin transport was severely inhibited by APP overexpression, and that synaptotagmin transport can depend on kinesin 3 [67], [68] and kinesin 1 (both KLC and kinesin 1 heavy chain) [69]–[73], we tested the possibility that *APP* overexpression perturbs KLC and synaptotagmin interaction via immunoprecipitation. APP overexpression indeed reduced synaptotagmin (cargo) and KLC interaction while overexpression of *APP* and *nebula* preserved this interaction (Fig. 10C). These results suggest that Nebula is likely to restore APP-induced axonal transport defects by correcting GSK-3β signaling and stabilizing cargo-motor interaction.

### Complex Interaction between Calcineurin and GSK-3β Signaling Regulates Axonal Transport

Having demonstrated that APP activates calcineurin signaling to regulate GSK-3β phosphorylation, we next examined if reducing GSK-3β can restore axonal transport. In the presence of APP upregulation, decreasing Shaggy (Sgg; fly homolog of GSK-3β) in flies with *APP* overexpression (*sgg^1^*;*APP*) resulted in significant suppression of the APP aggregate phenotype (Figs. 10D and 10E). This result is consistent with a recent report demonstrating mild enhancement of APP-YFP movement when GSK-3β is reduced [51]. Surprisingly, normal calcineurin activity was detected in these flies (1.00±0.16 fold of control for *Sgg^1^*;*APP* vs. 1.75±0.25 fold of control for APP). This result suggests the existence of feedback regulation of calcineurin activity and further implies that either a change in calcineurin activity or GSK-3β signaling could be responsible for the observed rescue. We therefore generated flies expressing *APP* and constitutively active calcineurin in *sgg^1^* background (*sgg^1^;APP/CaN^Act^*). Note that we used the hypomorphic allele *sgg^1^* because *sgg* null animals are lethal [74]. Consistent with GSK-3β being downstream of calcineurin, reducing Sgg diminished the effect of CaN^Act^ in enhancing APP phenotype (Figs. 9B and 10E). We also expressed the constitutively active Sgg (*sgg^S9A^*) together with *APP*, which surprisingly showed the same phenotype as *APP* overexpression. Calcineurin activity assay showed an unexpected decrease in calcineurin activity (0.74±0.06 fold of control) in these flies, suggesting that constitutive GSK-3β activation in the absence of calcineurin activation is sufficient to disrupt axonal transport potentially through phosphorylation of KLC. Interestingly, we find that overexpression of the constitutively active Sgg in neurons alone was sufficient to induce aggregate accumulation similar to flies with *APP* overexpression (Figs. 10D and 10E). Calcineurin activity assay revealed that these flies showed an increase in overall calcineurin activity (1.65±0.30 fold of control). This increase in calcineurin activity by active Sgg may be due to GSK dependent phosphorylation of Nebula, which has been shown to cause activation of calcineurin [75]. Since over-activation of calcineurin and GSK-3β pathway in the absence of APP upregulation fully replicated the aggregate accumulation phenotype, it suggests that abnormal activation of both the GSK-3β and calcineurin pathways are necessary for the severe axonal transport defect and aggregate accumulation phenotypes.

## Discussion

We have demonstrated a novel role for Nebula, the *Drosophila* ortholog of DSCR1, in ameliorating axonal transport impairments associated with the upregulation of APP. We find that Nebula upregulation significantly delayed photoreceptor neurodegeneration and dramatically decreased the axonal “traffic jam” phenotype caused by *APP* overexpression. Reducing Nebula independent of APP was sufficient to trigger defects in axonal transport, suggesting that Nebula is normally required for reliable delivery of synaptic cargos, likely through calcineurin dependent pathway. We demonstrate for the first time that *APP* overexpression causes calcineurin-dependent activation of GSK-3β kinase *in vivo*, thus implicating altered calcineurin signaling as a novel mechanism regulating axonal transport (Fig. S11). We find that co-upregulation of Nebula preserved the vesicular cargo to molecular motor interaction, ameliorated axonal transport defects, and protected against locomotor deficits. As impaired transport of essential organelles and synaptic vesicles caused by perturbation of APP is thought to precede synaptic failure and neurodegeneration in AD, our findings further suggest that DSCR1 upregulation may be a neuroprotective mechanism used by neurons to combat the effects of APP upregulation and delay progression of AD.

### Insights into Mechanisms Underlying APP-Induced Transport Defects

Although upregulation of APP had been shown to negatively influence axonal transport in mouse and fly models 28–31, mechanisms by which APP upregulation induces transport defects are poorly understood. Several hypotheses have been proposed, including titration of motor/adaptor by APP, impairments in mitochondrial bioenergetics, altered microtubule tracks, or aberrant activation of signaling pathways [76]. The motor/adaptor titration theory suggests that excessive APP-cargos titrates the available motors away from other organelles, thus resulting in defective transport of pre-synaptic vesicles [29]. Our finding that Nebula co-upregulation enhanced the movement and delivery of both synaptotagmin and APP to the synaptic terminal argues against this hypothesis. In addition, earlier finding suggest that Nebula upregulation alone impaired mitochondrial function and elevated ROS level [77], thus implying that Nebula is not likely to rescue APP-dependent phenotypes by selectively restoring mitochondrial bioenergetics. Furthermore, consistent with a recent report showing normal microtubule integrity in flies overexpressing either *APP-YFP* or activated *GSK-3β* [51], our data revealed normal gross microtubule structure in flies with *APP* overexpression. Together, these results suggest that changes in gross microtubule structure and stability is not a likely cause of APP-induced transport defects.

Instead, our results support the idea that Nebula facilitates axonal transport defects by correcting APP-mediated changes in phosphatase and kinase signaling pathways. First, we find that APP upregulation elevated intracellular calcium level and calcineurin activity, and that restoring calcineurin activity to normal suppressed the synaptotagmin aggregate accumulation in axons. The observed increase in calcium and calcineurin activity is consistent with reports of calcium dyshomeostasis and elevated calcineurin phosphatase activity found in AD brains [78]–[80], as well as reports demonstrating elevated neuronal calcium level due to *APP* overexpression and increased calcineurin activation in Tg2576 transgenic mice carrying the *APP^swe^* mutant allele [81], [82]. Second, APP upregulation resulted in calcineurin dependent dephosphorylation of GSK-3β at Ser9 site, a process thought to activate GSK-3β kinase [56]. APP upregulation also triggered calcineurin-independent phosphorylation at Tyr216 site, which has been shown to enhance GSK-3β activity [64], [65]. The kinase(s) that phosphorylates APP at Tyr216 is currently not well understood, it will be important to study how APP leads to Tyr216 phosphorylation in the future. Based on our results, we envision that *APP* overexpression ultimately leads to excessive calcineurin and GSK-3β activity, whereas *nebula* overexpression inhibits calcineurin to prevent activation of GSK-3β (Fig. S11). Our findings that *nebula* co-overexpression prevented GSK-3β activation and enhanced the transport of APP-YFP vesicles are consistent with a recent report by Weaver *et al.*, in which they find decreasing GSK-3β in fly increased the speed of APP-YFP movement [51]. Furthermore, consistent with our result that APP upregulation triggers GSK-3β enhancement and severe axonal transport defect, Weaver *et al.* did not detect changes in GFP-synaptotagmin movement in the absence of APP upregulation.

Active GSK-3β has been shown to influence the transport of mitochondria and synaptic proteins including APP, although the exact mechanism may differ between different cargos and motors [51], [83], [84]. One mechanism proposed for GSK-3β-mediated regulation of axonal transport is through phosphorylation of KLC1, thereby disrupting axonal transport by decreasing the association of the anterograde molecular motor with its cargos [59]. Accordingly, we find that APP reduced KLC-synaptotagmin interaction while Nebula upregulation preserved it. Synaptotagmin transport in both the anterograde and retrograde directions were affected, consistent with previous reports showing that altering either the anterograde kinesin or retrograde dynein is sufficient affected transport in both directions [85], [86]. Our results also support work suggesting that synaptotagmin can be transported by the kinesin 1 motor complex in addition to the kinesin 3/imac motor [67]–[73]. As kinesin 1 is known to mediate the movement of both APP and mitochondria [37], [86]–[88], and that phosphorylation of KLC had been shown to inhibit mitochondrial transport [89], detachment of cargo-motor caused by GSK-3β mediated phosphorylation of KLC may lead to general axonal transport problems as reported here. However, GSK-3β activation may also perturb general axonal transport by influencing motor activity or binding of motors to the microtubule tract. Interestingly, increased levels of active GSK-3β and phosphorylated KLC and dynein intermediate chain (DIC), a component of the dynein retrograde complex, have been observed in the frontal complex of AD patients [90]. Genetic variability for KLC1 is thought to be a risk factor for early-onset of Alzheimer's disease [91]. There is also increasing evidence implicating GSK-3β in regulating transport by modulating kinesin activity and exacerbating neurodegeneration in AD through tau hyperphosphorylation [21], [51], [55]. It will be interesting to investigate if Nebula also modulates these processes in the future.

### Calcineurin and GSK-3β Signaling Interactions

Although calcineurin had been shown to regulate many important cellular pathways, the link between altered calcineurin and axonal transport, especially in the context of AD, had not been established before. We show that calcineurin can regulate axonal transport through both GSK-3β independent and dependent pathways. This is supported by our observation that the severity of the aggregate phenotype was worse for flies expressing APP and active calcineurin than it was for flies expressing APP and active GSK-3β. These findings point to a role for calcineurin in influencing axonal transport directly, perhaps through dephosphorylation of motor or adaptor proteins. Our data also indicate that calcineurin in part modulates axonal transport through dephosphorylation of GSK-3β as discussed above; however, upregulation of APP is necessary for the induction of severe axonal transport problems, mainly by causing additional enhancement of GSK-3β signaling. GSK3 inhibition is widely discussed as a potential therapeutic intervention for AD, our results suggest that perhaps calcineurin is a more effective target for delaying degeneration by preserving axonal transport.

### Implications for Delayed Progression of AD in DS

DSCR1 and APP are both located on chromosome 21 and upregulated in DS [4], [10]. Overexpression of DSCR1 alone had been contradictorily implicated in both conferring resistance to oxidative stress and in promoting apoptosis [8], [9], [16], [17]. Upregulation of Nebula/DSCR1 had also been shown to negatively impact learning and memory in fly and mouse models through altered calcineurin pathways [15], [92]. How could upregulation of DSCR1 be beneficial? We propose that DSCR1 upregulation in the presence of APP upregulation compensates for the altered calcineurin and GSK-3β signaling, shifting the delicate balance of kinase/phosphatase signaling pathways close to normal, therefore preserving axonal transport and delaying neurodegeneration. We also propose that axonal transport defects and synapse dysfunction caused by APP upregulation in our *Drosophila* model system occur prior to accumulation of amyloid plaques and severe neurodegeneration, similar to that described for a mouse model [30].

DS is characterized by the presence of AD neuropathologies early in life, but most DS individuals do not exhibit signs of dementia until decades later, indicating that there is a delayed progression of cognitive decline [2], [93]. The upregulation of DSCR1 may in fact activate compensatory cell signaling mechanisms that provide protection against APP-mediated oxidative stress, aberrant calcium, and altered calcineurin and GSK3-β activity.

## Materials and Methods

### Fly Stocks

Flies were cultured at 25°C on standard cornmeal, yeast, sugar, and agar medium under a 12 hour light and 12 hour dark cycle. The following fly lines were obtained from the Bloomington Drosophila Stock Center: *Gmr-GAL4*, *UAS-APP695-N-myc* (6700), *sgg^1^/FM7a, UAS-sgg^S9A^* (Sgg constitutively active), *UAS-nla-RNAi* (27260), *UAS-CaNB-RNAi* (27307), *UAS-syt.eGFP* (6925), *UAS-APP.YFP* (32039), and *UAS-mitoGFP*. *Elav-GAL4* stock was kindly provided by Dr. Feany (Harvard University), *UAS-nla^t1^*, and *nla^1^* flies were reported previously [15]. *UAS-ΔCaN^Act^* construct (constitutively active calcineurin) was generated by deleting the autoinhibitory domain of the CaNA gene Pp2B-14D and subcloned into the pINDY6 vector similar to that described [94]. UAS-Case12 was generated by inserting Case12 (from Evrogen) into pINDY6 vector [53]. Transgenic flies were generated by standard germline transformation method [95].

### Histology

Adult *Drosophila* of 0, 15, 30 and 45 days of age were collected, decapitated and had their proboscis removed. Heads were incubated in Mirsky's fixative for 30 minutes, washed with PBS, and post-fixed in 4% paraformaldehyde for 20 minutes. Fly heads were then transferred to 25% sucrose overnight at 4°C and were subsequently embedded in Tissue-Tek O.C.T Compound for cryostat sectioning (10 µm). Photoreceptor axons were immunostained with 24B10 (1∶10; Developmental Studies Hybridoma), Phosphorylated APP (1∶400; Sigma), and 4G8 (1∶500; Signet).

### Phototaxis

Flies were placed in 2 clear round bottom test tubes joined at the opening. After allowing 2 minutes for the flies to acclimate to the tubes, flies were lightly tapped and the percentage of flies that moved toward light in horizontal position within 30 seconds was counted.

### Immunocytochemistry

Wandering 3^rd^ instar larvae were dissected in cold calcium-free dissection buffer and fixed with 4% paraformaldehyde in PBS for 25 minutes at room temperature (RT). Samples were blocked in 5% normal goat serum in PBS+0.1% triton for 1 hour at RT and then incubated with primary antibodies overnight at 4°C. Antibodies included synaptotagmin (1∶1,000; gift from H. Bellen) and mAb 4G8 (1∶1,000; Signet), β-tubulin (1∶1000; DSHB), acetylated tubulin (1∶500, Abcam), Cy3-conjugated HRP (1∶200, Jackson ImmunoResearch). Alexa-conjugated secondary antibodies were applied at 1∶500 and samples mounted in Pro -long Gold Antifade reagent (Invitrogen).

### Static Image Acquisition and Quantification

Images of motor axons and synaptic terminals from NMJ 6/7 in segment A2 or A3 were captured in a z-series using Zeiss LSM5 scanning confocal. The number of aggregates was determined manually by counting the number of punctate staining with intensity above background and size greater than 0.2 µm^2^. For quantification of antibody staining intensities at the NMJ, dissected larvae were stained together using the same condition. Images were captured in a z-series and parameters were set to minimize saturation of pixel intensity. Intensity of Z-projected images was analyzed using ImageJ and fold changed calculated by comparing to the control.

### Live-Imaging

Wandering 3^rd^ instar larvae expressing APP-YFP or GFP-SYT in combination with other transgenes were dissected in calcium free dissection buffer: 128 mM NaCl, 1 mM EGTA, 4 mM MgCl_2_, 2 mM KCl, 5 mM HEPES, and 36 mM sucrose. Live imaging of GFP-SYT was done as described [96]. For imaging of mito-GFP, dissected larvae were bathed in HL-3 solution [97]. Time-lapse images were acquired at 5-s intervals using a Zeiss LSM5 confocal using minimum laser intensity to prevent photobleaching and damage to the tissues. Images were acquired for 5 minutes with a 63× lens and a zoom of 1.7. All live imaging experiments were completed within 15 minutes starting from the time of dissection in order to ensure health of the samples.

### Live Imaging Analysis

The Manual tracking Plugin in ImageJ was used to track individual vesicle and mitochondria movement. At least 10 frames (>50 s) were used to calculate the average speed of movement. Percentage of movement was determined by counting the percentage of moving vesicles over the imaging period. A vesicle is labeled as moving if it moved in three consecutive frames (over a 15-s period) over a distance of at least 0.1 µm. Direction of movement is determined by direction of net displacement of the vesicle at the start of imaging. Average speed was determined by tracking a vesicle for an uninterrupted run in either the anterograde or retrograde direction. The total distance of movement was divided by the total duration of movement in a specific direction. Student's t-test was used to determine statistical significance.

### Line Crossing Locomotor Assay

Deficits in larval locomotor behavior were assessed as described previously [98]. Briefly, larvae were washed with PBS and placed in 60 mm petri dish filled with 1% agarose. Using a moistened paint brush, 3^rd^ instar larvae were collected and allowed to habituate for 30 seconds. The number 0.5 cm^2^ boxes entered was counted for a 60-s period.

### Western Blots

*Drosophila* adults (1–2 days) were collected on dry ice. Heads were removed and homogenized in cold RIPA buffer. The brains of 3^rd^ instar larvae were dissected and collected on dry ice. Equal amount of protein per genotype (10–20 µg) was run on SDS polyacrylamide gel and transferred to nitrocellulose membrane. Blocking for phosphorylated antibodies was performed using 5% BSA in PBS+0.1% tween (PBS-TW). Blocking for non-phosphorylated antibodies was done using 5% milk in PBS-TW for one hour at RT. Membranes were incubated with the following primary antibodies overnight at 4°C: N-APP (1∶5,000; Sigma), β-tubulin (1∶500; Developmental Studies Hybridoma Bank), Nebula (1∶7,000), Fasciclin II (1∶50 Developmental Studies Hybridoma), acetylated tubulin (1∶1,000, Cell Signaling), phospho-GSK3β Ser9 (1∶1000, Cell Signaling), phospho-GSK3β Tyr126 (1∶1000, Cell Signaling), and GSK3 α/β (1∶2,000, Cell Signaling). Secondary antibodies used were: anti-mouse Alexa 680 (Invitrogen), anti-rabbit Dylight 800 (Piercenet), anti-mouse coupled HRP or anti-rabbit coupled HRP. HRP signals were detected using ECL Reagents (GE Healthcare). Alexa 680 and Dylight 800 signals were detected using Odyssey Imaging system (LI-COR Biosciences). For reprobing, membranes were stripped using Reblot Plus strong antibody stripping solution (Millipore) and reprobed. NIH Image J software was used to measure signal intensity, and the fold change in specific protein level was normalized to a loading control and compared to the control flies.

### Calcineurin Activity

Fly heads were collected over dry ice, decapitated, and homogenized in lysis buffer (10 mM Tris pH 7.5, 1 mM EDTA, 0.02% Sodium Azide). Calcineurin phosphatase activity was determined using the Ser/Threonine Phosphatase Assay Kit (Promega) following the manufacturer's protocol as done previously [15]. 5 µg of protein per genotype was used.

### Immunoprecipitation

Flies heads were collected on dry ice by passing through molecular sieves and homogenized in lysis buffer (10 mM HEPES, 0.1 M NaCl, 1% NP-40, 2 mM EDTA, 50 mM NaF, 1 mM NA_3_VO_4_) plus Complete Mini protease inhibitor cocktail (Roche). Lysates were pre-cleared by incubating fly extract with magnetic A/G beads (Thermo Scientific) for 1 hour at 4°C. Pre-cleared extract was then used for IP using GFP antibody conjugated to magnetic beads (MBL International). Western blot analysis using an antibody against the kinesin light chain (1∶200; Novus Biologicals) was used to confirm interaction. To determine the efficiency of GFP pull down, an antibody against GFP (1∶1000, Abnova) was also used. To eliminate signal contamination from IgG, we used HRP conjugated TrueBlot anti-rabbit IgG (1∶1000, ebioscience) that is specific for native IgG as secondary antibody.

## Supporting Information

Figure S1 Levels of APP and Nebula driven by Gmr-GAL4. (A) Western blots depicting the levels of APP and Nebula in flies overexpressing the indicated transgenes using the *Gmr-GAL4* driver. Control flies carry one copy of *UAS-LacZ* gene driven by *Gmr-Gal4*. To normalize the number of transgenes found in different fly lines, *UAS-LacZ* was crossed into the background of flies with *APP* overexpression (OE), or *APP*;*nla^1^* flies. Note that transgenic line *nla^t1^* contains *nebula* transgene tagged with HA, and hence the overexpressed Nebula appears as a higher band. (B) Quantification of the relative protein levels for the indicated fly lines. Values represent mean ± SEM, n = 4 independent experiments. * P<0.05 compared to control. All calculations were normalized to loading control, β-tubulin.
(TIF)

Figure S2 Levels of Nebula in the indicated fly lines. (A) Western blots showing the level of Nebula in *nla^1^* mutant (containing one copy of *Elav-Gal4* so that it is in same genetic background), and *RNAi-nla* driven by the neuronal *Elav-Gal4* driver. (B) Quantification of Nebula protein level. Values represent mean ± SEM, n = 3 independent experiments. * P<0.05 compared to control. All calculations were normalized to loading control, β-tubulin.
(TIF)

Figure S3 Levels of APP and Nebula in the indicated transgenic lines driven by the pan-neuronal *Elav-GAL4* driver. (A) Western blots depicting the levels of APP and Nebula in fly heads overexpressing the indicated transgenes using the *Elav-Gal4* driver. Protein loading level is indicated by β-tubulin. Because transgenic line *nla^t1^* contains *nebula* transgene tagged with HA, the overexpressed Nebula protein appears as a higher band. (B) Quantification of APP and Nebula proteins in fly head extracts. Values represent mean ± SEM, n = 4 independent experiments. * P<0.05 compared to control. All calculations were normalized to loading control, β-tubulin.
(TIF)

Figure S4 Levels of APP and Nebula in the brains of 3^rd^ instar larvae. (A) Western blot depicting the level of APP in larvae overexpressing the indicated transgenes. All transgenes were driven by the neuronal *Elav-Gal4* driver. Lower graph shows quantification of APP protein level in dissected larval brains. Relative values depicted in comparison to APP. (B) Western blot depicting the level of Nebula in larval brain extracts. Lower graph shows quantification of Nebula protein level. All values represent mean ± SEM, n≥3 independent experiments. All calculations were normalized to loading control, β-tubulin. * P<0.05 compared to control.
(TIF)

Figure S5 Nebula reduction decreases synaptotagmin delivery to the neuromuscular junction (NMJ) and causes locomotor deficits. (A) Synaptotagmin (SYT) staining in the segmental motor axons. (B) Quantification of SYT aggregate number and protein level in the NMJ. n = 6 independent experiments. (C) Western blots showing that the level of overall SYT level was not altered. (D) SYT staining in the NMJ for the indicated genotypes. Right panels show pseudo colored SYT staining and intensity scale. (E) Locomotor assay. n = 10 independent experiments. For (B) and (E), values represent mean ± SEM * indicates P<0.05 compared to control. Scale bars = 10 µm.
(TIF)

Figure S6 Nebula modulates *Drosophila* APPL-induced transport deficits in a similar fashion to human APP. (A) Synaptotagmin (SYT) staining in the NMJ for the indicated genotypes. The *APPL* overexpression lines were driven by the pan-neuronal Elav-Gal4 driver and the *Appl^d^;RNAi-nla* line was driven by the pan-neuronal *nSyb-GAL4* driver. Right panels show pseudo-colored SYT staining and intensity scale. (B) Quantification of SYT level in the NMJ normalized to the control. Values represent the mean ± SEM, n = 6 independent experiments * indicates P<0.05 compared to control unless otherwise indicated. (C) SYT staining in the axonal nerves of the *Appl^d^* lines. Scale bars = 10 µm.
(TIF)

Figure S7 Nebula co-overexpression increases delivery of Fasciclin to the synaptic terminal. (A) Pseudo-colored images (left column) of Fasciclin (FasII) staining in the NMJ of 3^rd^ instar larvae (A2 of muscle 6/7). Right panels show FasII (green) and HRP staining (red) outlining the synaptic bouton structure. Scale bar = 10 µm. (B) Quantification of the relative intensity of FasII in the terminal normalized to the control. Values represent mean ± S.E.M, * p≤0.05 compared to control unless otherwise indicated, n≥5 independent experiments per genotype.
(TIF)

Figure S8 *APP* overexpression does not significantly alter distribution of mitochondria. (A) Quantification of the number of mitochondria normalized to the length of the nerve. (B) APP overexpression did not cause accumulation of mitochondria near sites of SYT aggregates (red). To determine distribution of mitochondria, mitochondrial targeted GFP (mito-GFP) was expressed together with the indicated transgenes. Scale bar = 10 µM. n≥6 independent experiments and all values represent mean ± SEM.
(TIF)

Figure S9 Modulation of APP-induced phenotypes by calcineurin. (A) Diagram of the constitutively active calcineurin construct (*CaN^Act^*) with calmodulin and autoinhibitory domain (AID) deleted. (B) Calcineurin activity. *CaN^Act^* indicates flies with transgene only but no driver, and *CaN^Act^* OE indicates *CaN^Act^* overexpression in neurons. n = 4 assays. (C) Images showing NMJs stained with SYT (green; bottom panels). Upper panels are pseudo-colored images with intensity scale shown on the right. (D) Quantification of SYT level in the NMJ. n>6 independent experiments. (E) Locomotor activity. n = 10 independent experiments. All values represent mean ± SEM. * indicates P<0.05 compared to control and ** P<0.05 compared to the indicated genotype.
(TIF)

Figure S10 Calcimycin application increases the fluorescence intensity of Case12 signal in fly neurons. (A) DIC image of the larval brain (left) highlighting the region imaged (right). Fluorescence intensity was determined before and after calcimycin treatment. (B) Quantification of the relative fluorescent intensity before and after calcimycin addition. n = 3 independent experiments. All values represent mean ± SEM * indicates P<0.05 compared to control.
(TIF)

Figure S11 Graphical representation of the interactions between APP, Nebula, calcineurin, and GSK-3β. (A) The left-hand panel depicts downstream signaling events arising from APP upregulation. *APP* overexpression increases intracellular calcium level which activates calcineurin (CaN) activity and an unknown pathway that leads to phosphorylation of GSK-3β at Y216 (or Y214 in *Drosophila*). Phosphorylation of GSK-3 atβ Y216 is required for enhancement of GSK-3β activity. Active calcineurin also dephosphorylates GSK-3β at Ser9 to relieve inhibition of GSK-3β, leading to activation of GSK-3β with enhanced activity. GSK-3β activation strongly contributes to axonal transport problems through downstream pathways, whereas calcineurin (independent of GSK-3β pathway) weakly contributes to axonal transport problems. APP upregulation therefore impairs axonal transport through two independent but interacting signaling pathways. (B) The right-hand panel introduces the downstream signaling events arising from interactions between APP and Nebula co-upregulation. Nebula inhibits calcineurin activity, thereby prevents activation of GSK-3β by calcineurin. Even though APP still triggers GSK-3β phosphorylation at Y216, phosphorylation at Ser9 overrides and inhibits activation of GSK-3β. Nebula inhibition of calcineurin rescues APP-mediated axonal transport defects by restoring both GSK-3β and calcineurin activity.
(TIF)

Movie S1 Video clip showing relative movement of larvae overexpressing either *APP* alone (APP OE) or *APP* together with *nebula* transgene (APP+Nla OE). Larvae were placed on agarose plate, habituated for 30 s and imaged for 1 minute. Video is shown at 8.5 frames per second. Note that towards the end of the movie, another App+Nla OE larva had moved into the field of view (lower right hand corner).
(AVI)

Movie S2 Nebula protects against APP-induced mitochondrial transport defects. Segmental nerves of 3^rd^ instar larvae expressing *mito-GFP* in neurons of control, flies overexpressing *APP* (*APP OE*) or *APP* with *nebula* (*APP+Nla OE*) were imaged for 5 minutes. Images were acquired at 5 s intervals, compressed 15 frames per section in the movie.
(AVI)
