# Supplementary figures and images for: Nebula/DSCR1 Upregulation Delays Neurodegeneration and Protects against APP-Induced Axonal Transport Defects by Restoring Calcineurin and GSK-3β Signaling
Source: PLoS Genet. 2013 Sep 26;9(9):e1003792. doi: 10.1371/journal.pgen.1003792 (PMC3784514; doi:10.1371/journal.pgen.1003792)

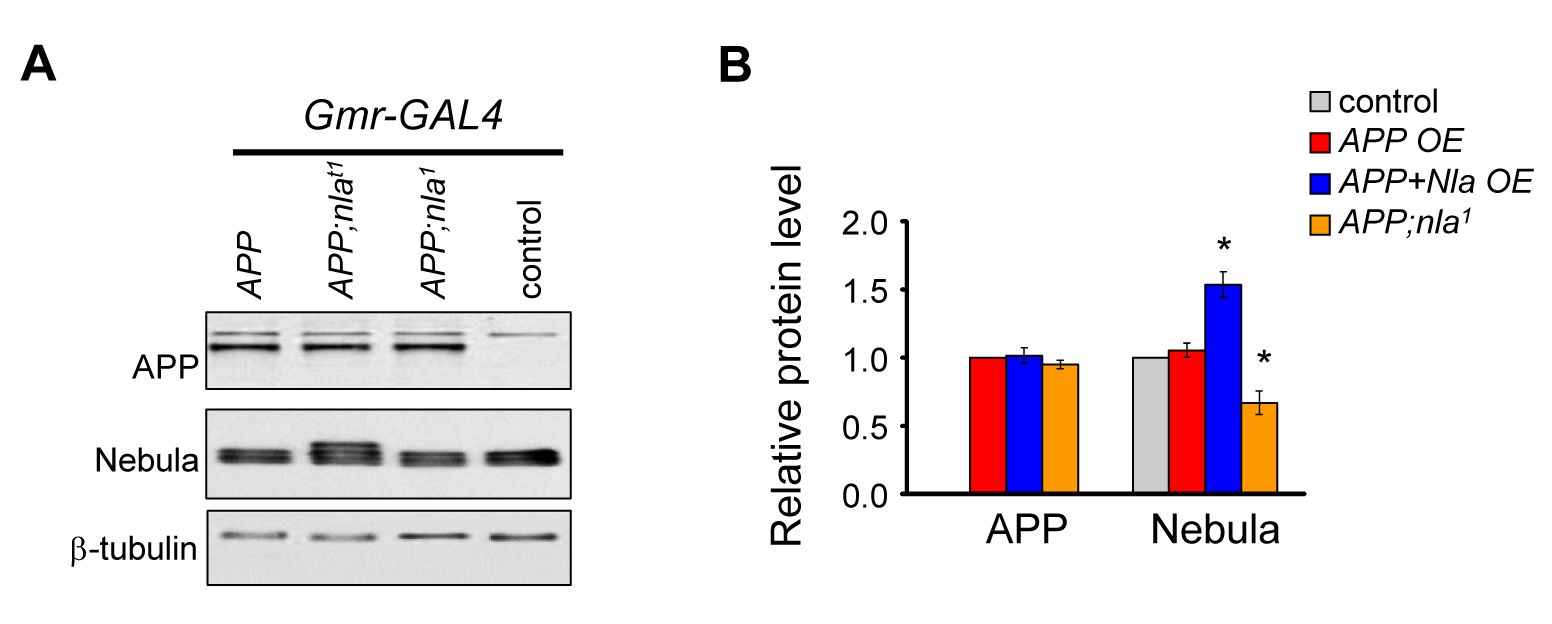

Supplement: Figure S1 — Levels of APP and Nebula driven by Gmr-GAL4. (A) Western blots depicting the levels of APP and Nebula in flies overexpressing the indicated transgenes using the Gmr-GAL4 driver. Control flies carry one copy of UAS-LacZ gene driven by Gmr-Gal4. To normalize the number of transgenes found in different fly lines, UAS-LacZ was crossed into the background of flies with APP overexpression (OE), or APP;nla1 flies. Note that transgenic line nlat1 contains nebula transgene tagged with HA, and hence the overexpressed Nebula appears as a higher band. (B) Quantification of the relative protein levels for the indicated fly lines. Values represent mean ± SEM, n = 4 independent experiments. * P<0.05 compared to control. All calculations were normalized to loading control, β-tubulin. (TIF) [file pgen.1003792.s001.tif]

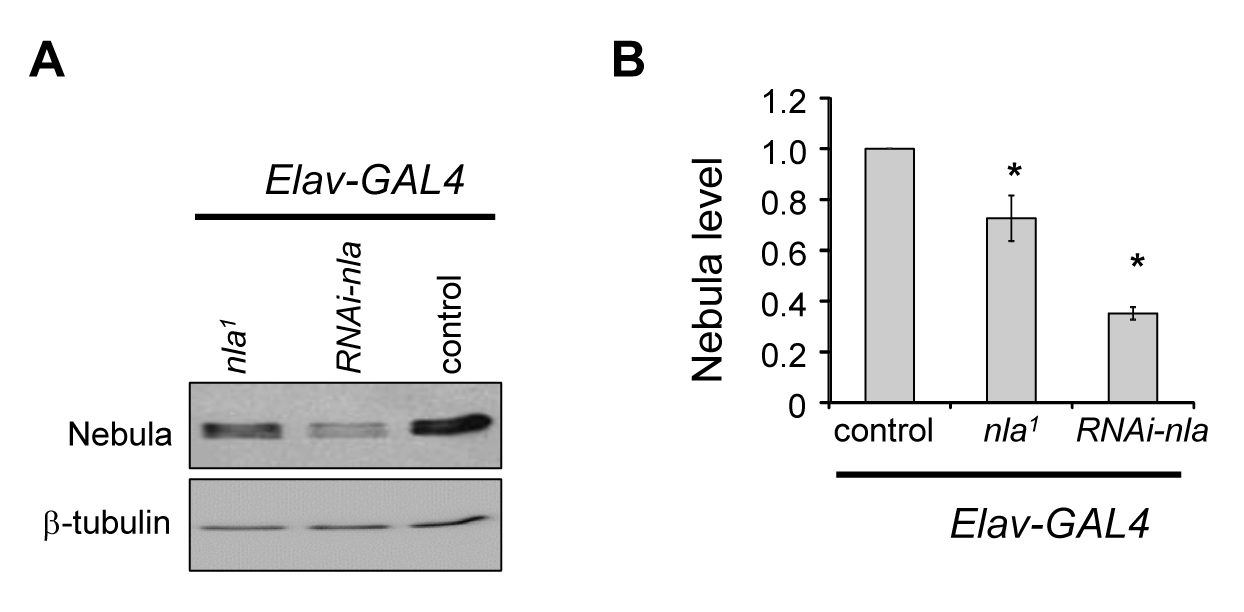

Supplement: Figure S2 — Levels of Nebula in the indicated fly lines. (A) Western blots showing the level of Nebula in nla1 mutant (containing one copy of Elav-Gal4 so that it is in same genetic background), and RNAi-nla driven by the neuronal Elav-Gal4 driver. (B) Quantification of Nebula protein level. Values represent mean ± SEM, n = 3 independent experiments. * P<0.05 compared to control. All calculations were normalized to loading control, β-tubulin. (TIF) [file pgen.1003792.s002.tif]

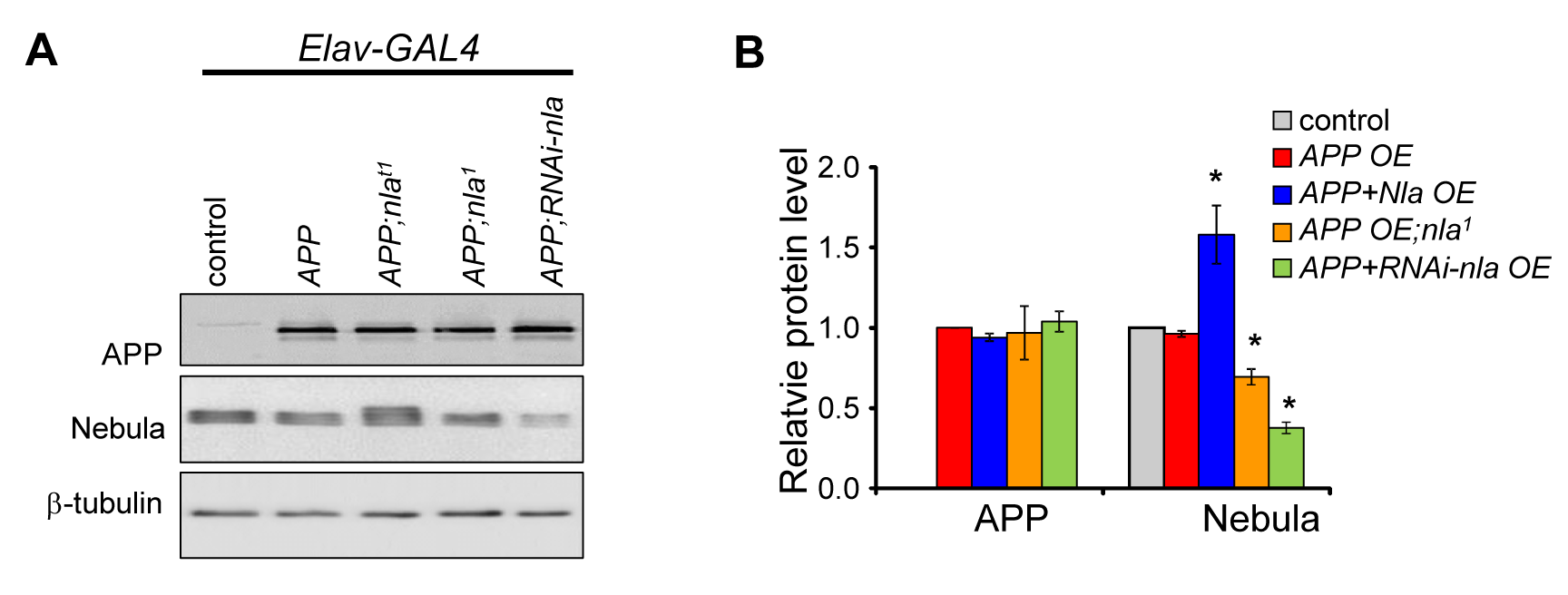

Supplement: Figure S3 — Levels of APP and Nebula in the indicated transgenic lines driven by the pan-neuronal Elav-GAL4 driver. (A) Western blots depicting the levels of APP and Nebula in fly heads overexpressing the indicated transgenes using the Elav-Gal4 driver. Protein loading level is indicated by β-tubulin. Because transgenic line nlat1 contains nebula transgene tagged with HA, the overexpressed Nebula protein appears as a higher band. (B) Quantification of APP and Nebula proteins in fly head extracts. Values represent mean ± SEM, n = 4 independent experiments. * P<0.05 compared to control. All calculations were normalized to loading control, β-tubulin. (TIF) [file pgen.1003792.s003.tif]

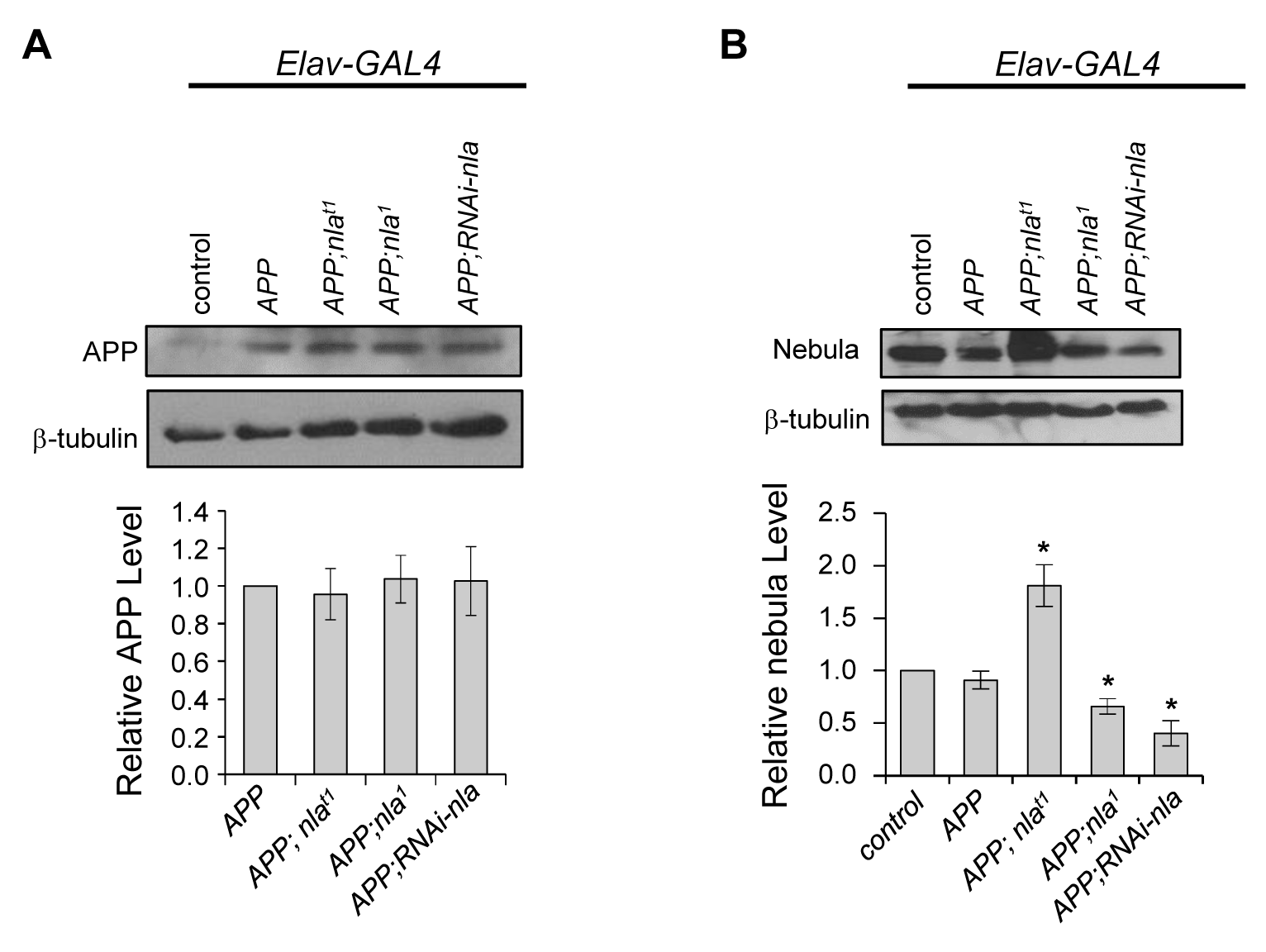

Supplement: Figure S4 — Levels of APP and Nebula in the brains of 3rd instar larvae. (A) Western blot depicting the level of APP in larvae overexpressing the indicated transgenes. All transgenes were driven by the neuronal Elav-Gal4 driver. Lower graph shows quantification of APP protein level in dissected larval brains. Relative values depicted in comparison to APP. (B) Western blot depicting the level of Nebula in larval brain extracts. Lower graph shows quantification of Nebula protein level. All values represent mean ± SEM, n≥3 independent experiments. All calculations were normalized to loading control, β-tubulin. * P<0.05 compared to control. (TIF) [file pgen.1003792.s004.tif]

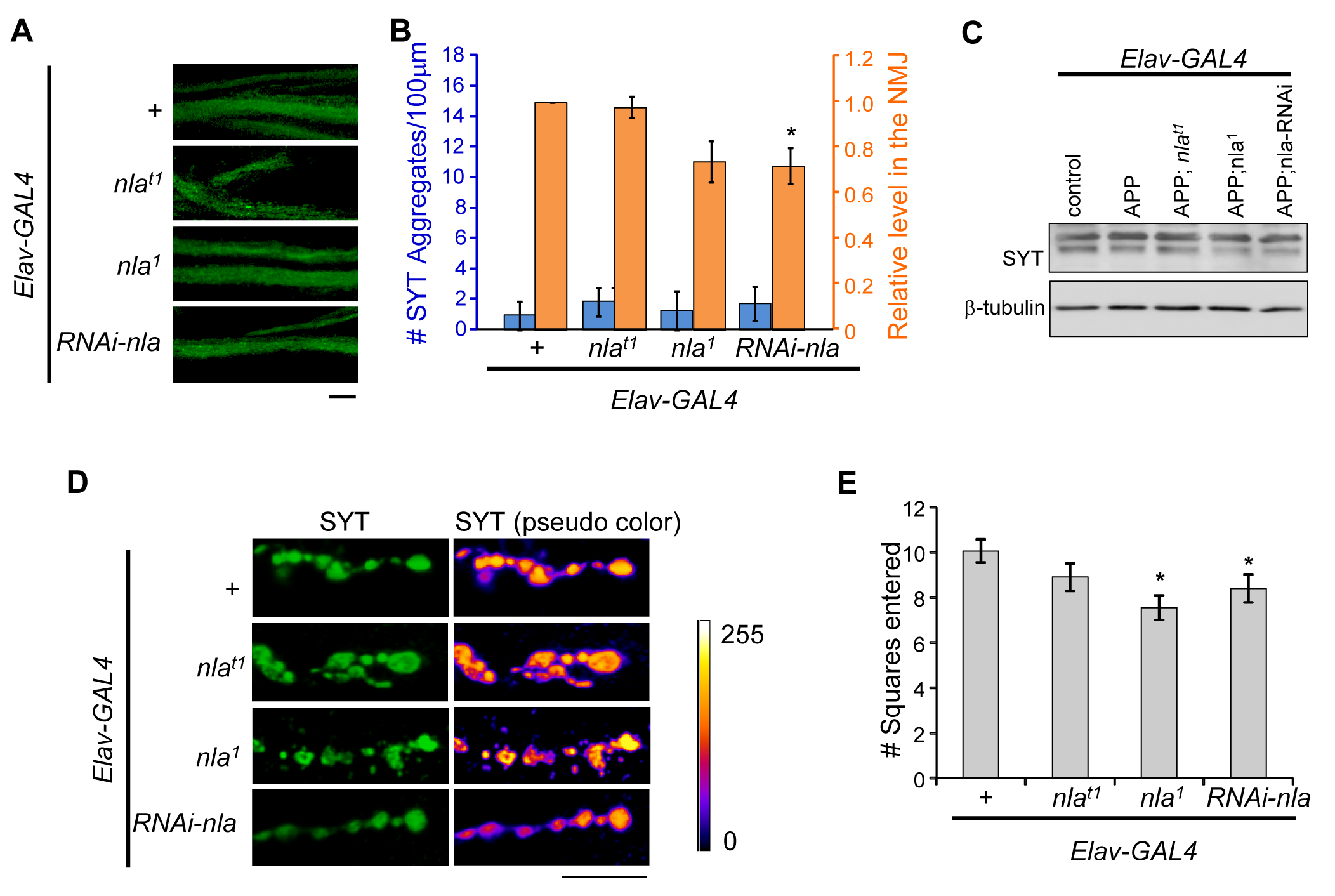

Supplement: Figure S5 — Nebula reduction decreases synaptotagmin delivery to the neuromuscular junction (NMJ) and causes locomotor deficits. (A) Synaptotagmin (SYT) staining in the segmental motor axons. (B) Quantification of SYT aggregate number and protein level in the NMJ. n = 6 independent experiments. (C) Western blots showing that the level of overall SYT level was not altered. (D) SYT staining in the NMJ for the indicated genotypes. Right panels show pseudo colored SYT staining and intensity scale. (E) Locomotor assay. n = 10 independent experiments. For (B) and (E), values represent mean ± SEM * indicates P<0.05 compared to control. Scale bars = 10 µm. (TIF) [file pgen.1003792.s005.tif]

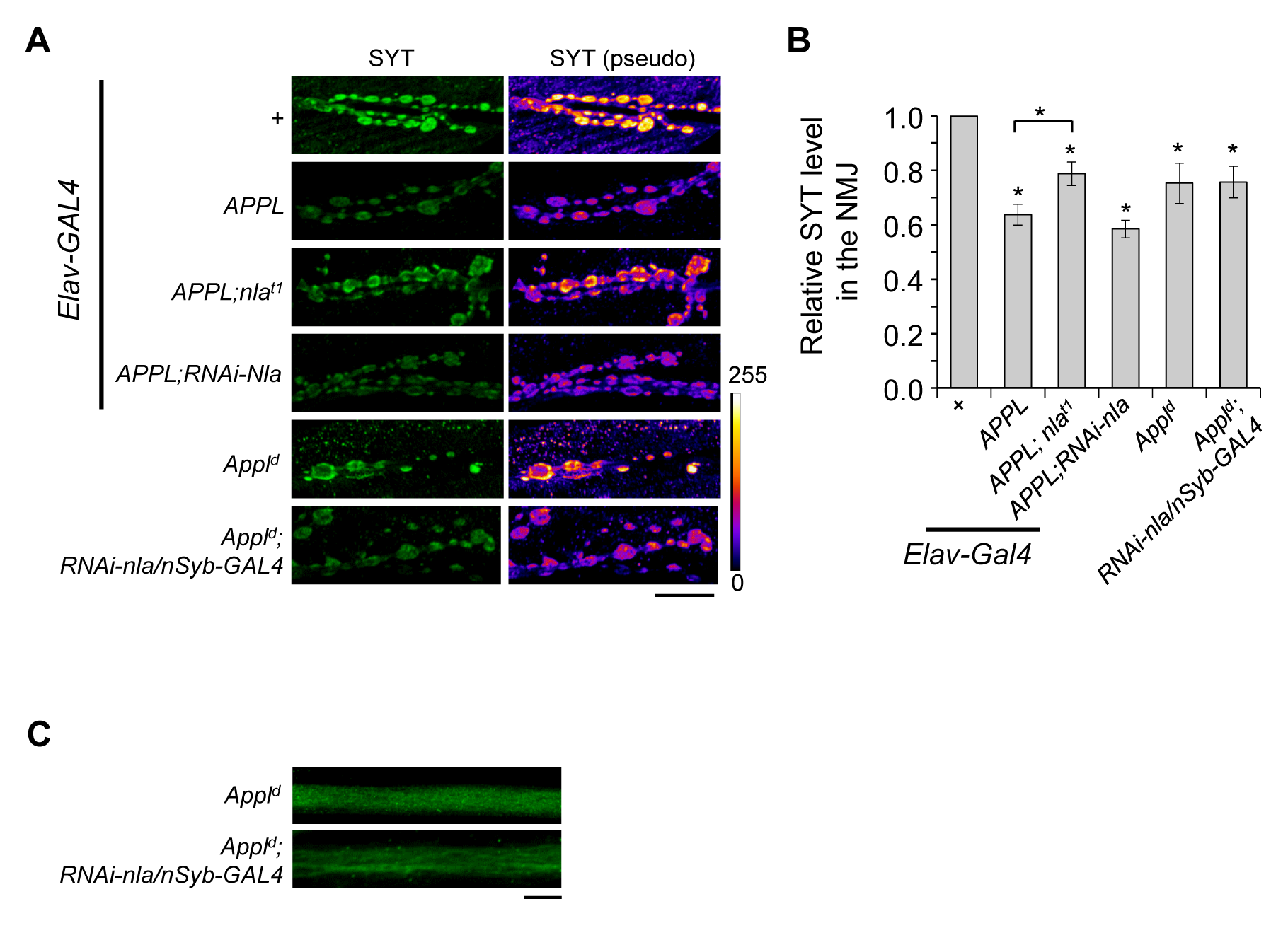

Supplement: Figure S6 — Nebula modulates Drosophila APPL-induced transport deficits in a similar fashion to human APP. (A) Synaptotagmin (SYT) staining in the NMJ for the indicated genotypes. The APPL overexpression lines were driven by the pan-neuronal Elav-Gal4 driver and the Appld;RNAi-nla line was driven by the pan-neuronal nSyb-GAL4 driver. Right panels show pseudo-colored SYT staining and intensity scale. (B) Quantification of SYT level in the NMJ normalized to the control. Values represent the mean ± SEM, n = 6 independent experiments * indicates P<0.05 compared to control unless otherwise indicated. (C) SYT staining in the axonal nerves of the Appld lines. Scale bars = 10 µm. (TIF) [file pgen.1003792.s006.tif]

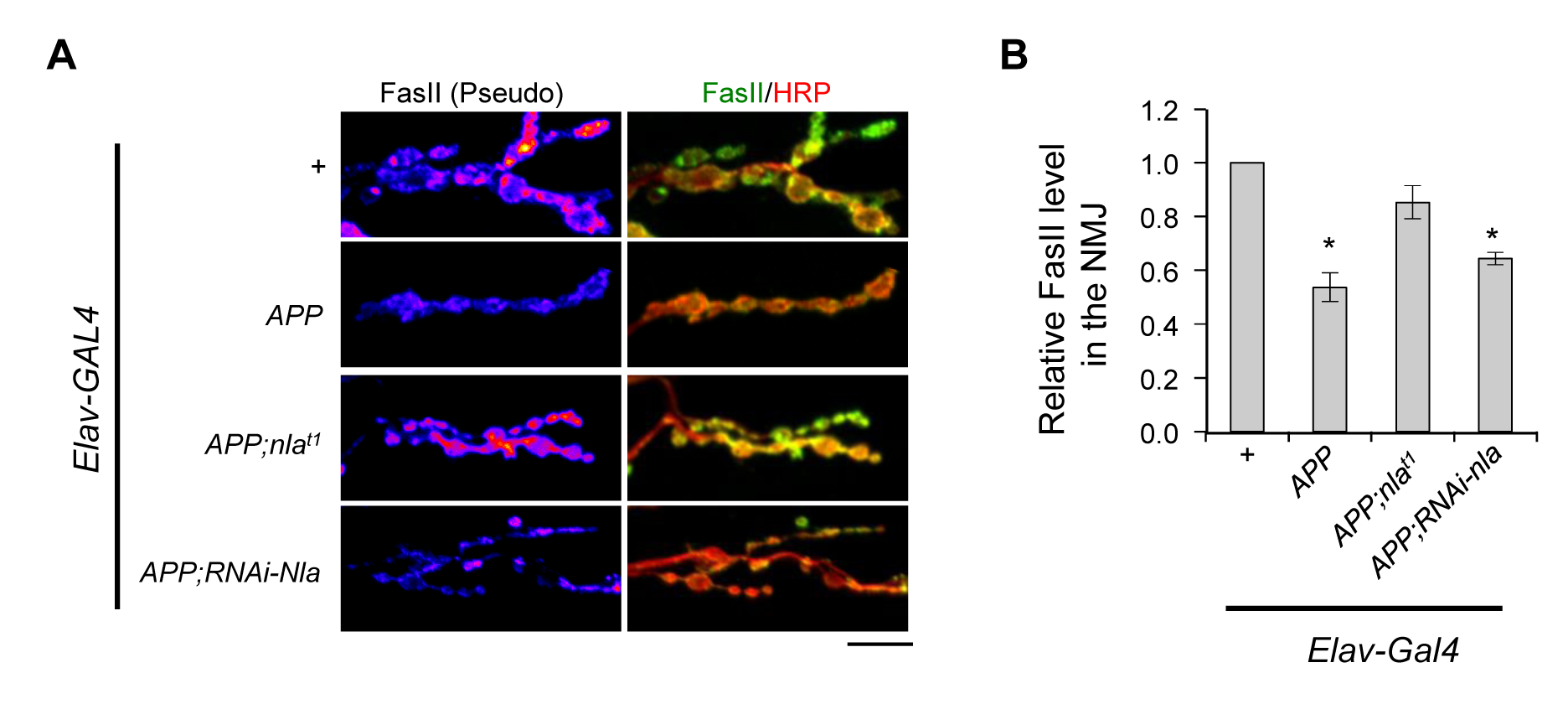

Supplement: Figure S7 — Nebula co-overexpression increases delivery of Fasciclin to the synaptic terminal. (A) Pseudo-colored images (left column) of Fasciclin (FasII) staining in the NMJ of 3rd instar larvae (A2 of muscle 6/7). Right panels show FasII (green) and HRP staining (red) outlining the synaptic bouton structure. Scale bar = 10 µm. (B) Quantification of the relative intensity of FasII in the terminal normalized to the control. Values represent mean ± S.E.M, * p≤0.05 compared to control unless otherwise indicated, n≥5 independent experiments per genotype. (TIF) [file pgen.1003792.s007.tif]

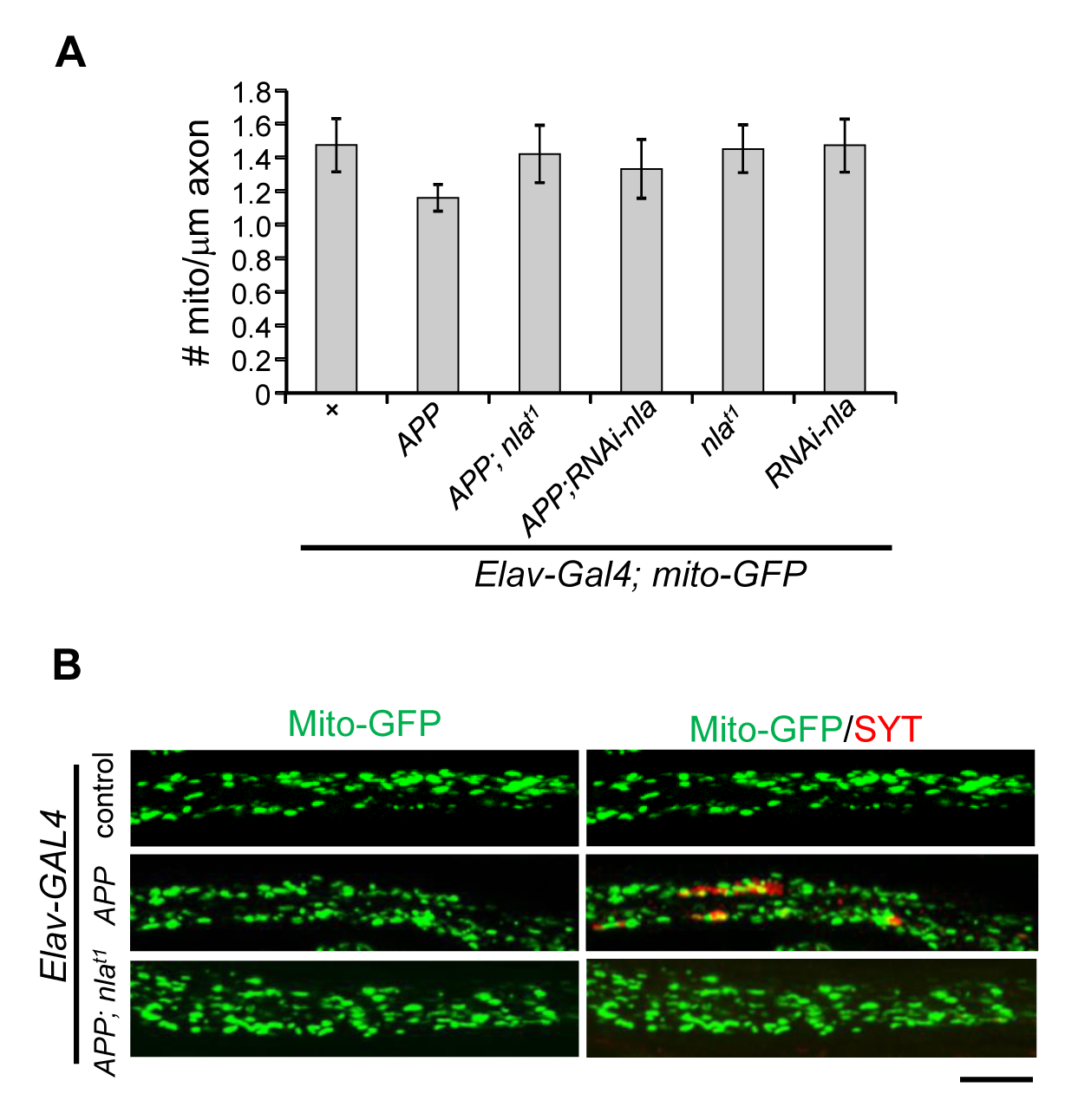

Supplement: Figure S8 — APP overexpression does not significantly alter distribution of mitochondria. (A) Quantification of the number of mitochondria normalized to the length of the nerve. (B) APP overexpression did not cause accumulation of mitochondria near sites of SYT aggregates (red). To determine distribution of mitochondria, mitochondrial targeted GFP (mito-GFP) was expressed together with the indicated transgenes. Scale bar = 10 µM. n≥6 independent experiments and all values represent mean ± SEM. (TIF) [file pgen.1003792.s008.tif]

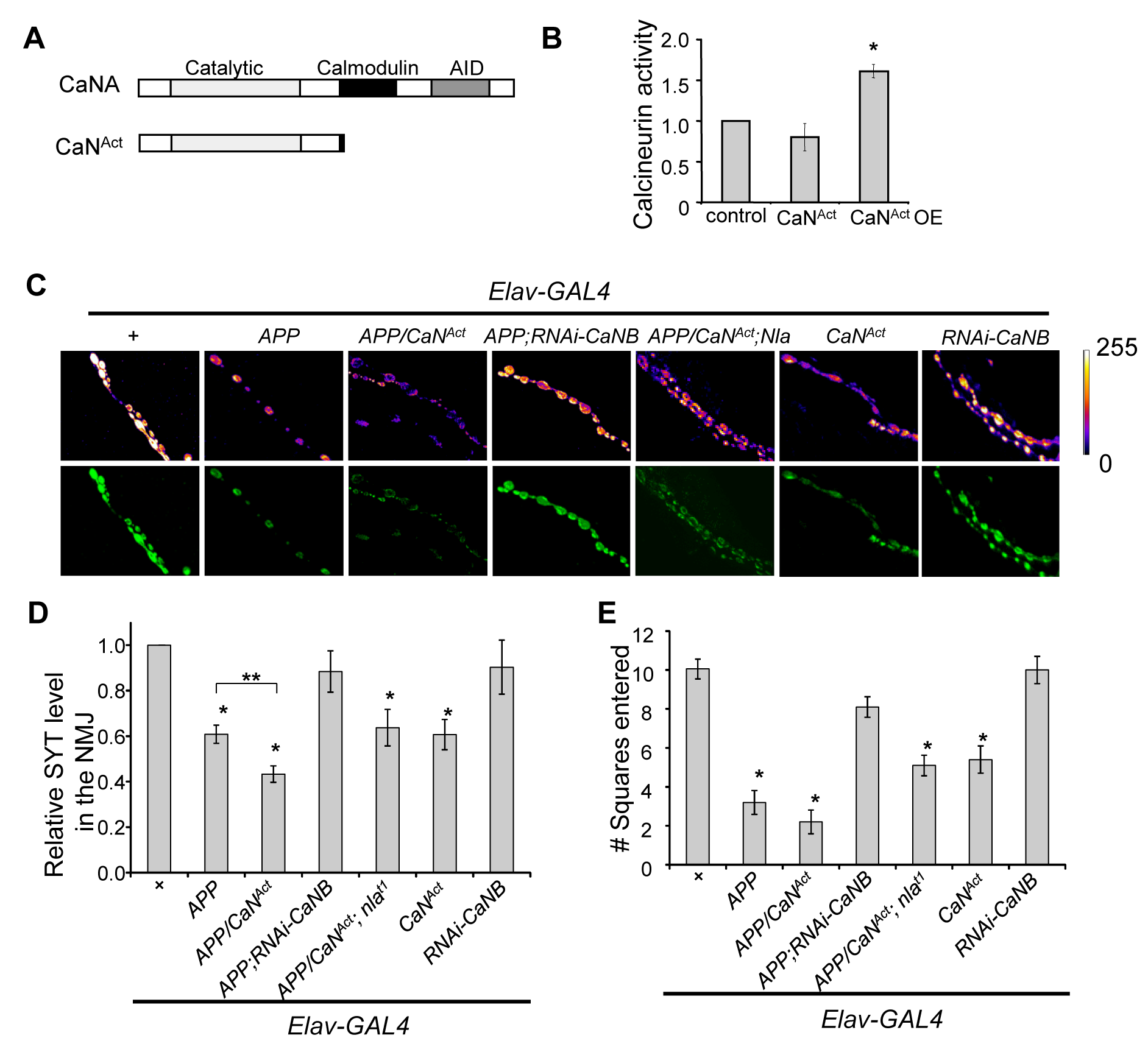

Supplement: Figure S9 — Modulation of APP-induced phenotypes by calcineurin. (A) Diagram of the constitutively active calcineurin construct (CaNAct) with calmodulin and autoinhibitory domain (AID) deleted. (B) Calcineurin activity. CaNAct indicates flies with transgene only but no driver, and CaNAct OE indicates CaNAct overexpression in neurons. n = 4 assays. (C) Images showing NMJs stained with SYT (green; bottom panels). Upper panels are pseudo-colored images with intensity scale shown on the right. (D) Quantification of SYT level in the NMJ. n>6 independent experiments. (E) Locomotor activity. n = 10 independent experiments. All values represent mean ± SEM. * indicates P<0.05 compared to control and ** P<0.05 compared to the indicated genotype. (TIF) [file pgen.1003792.s009.tif]

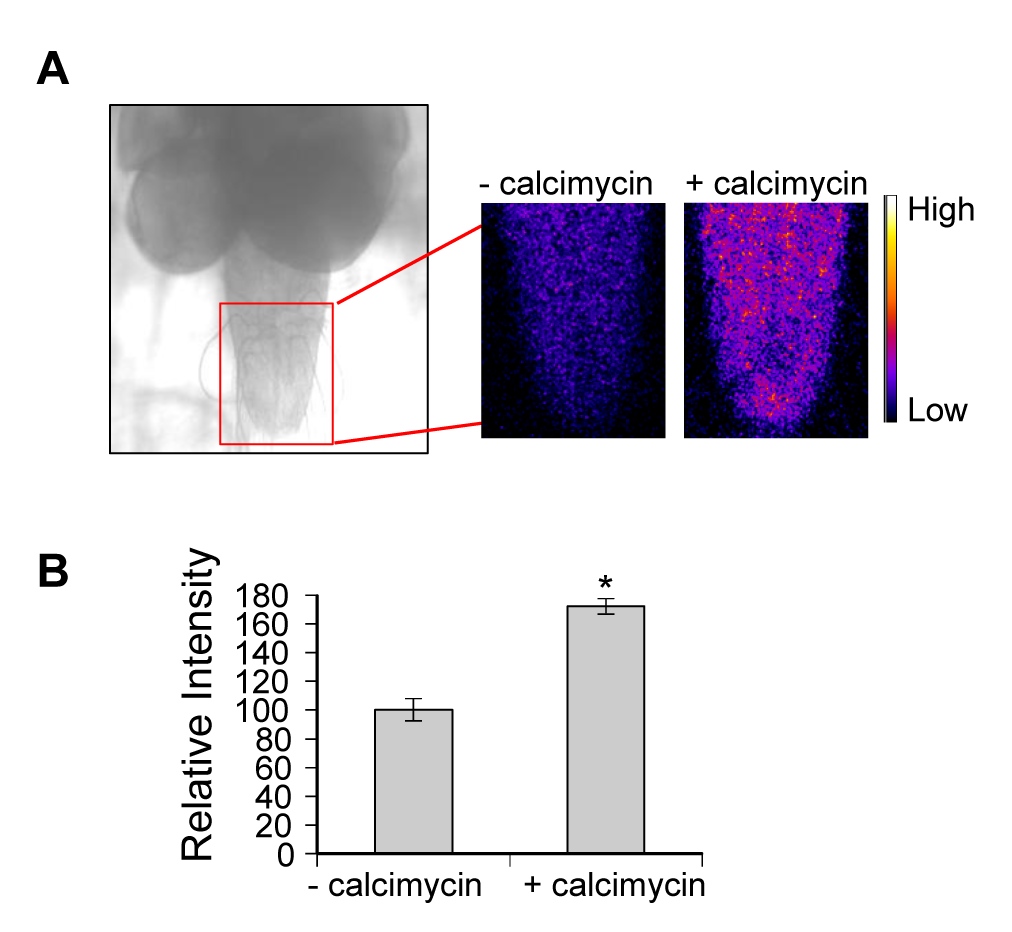

Supplement: Figure S10 — Calcimycin application increases the fluorescence intensity of Case12 signal in fly neurons. (A) DIC image of the larval brain (left) highlighting the region imaged (right). Fluorescence intensity was determined before and after calcimycin treatment. (B) Quantification of the relative fluorescent intensity before and after calcimycin addition. n = 3 independent experiments. All values represent mean ± SEM * indicates P<0.05 compared to control. (TIF) [file pgen.1003792.s010.tif]

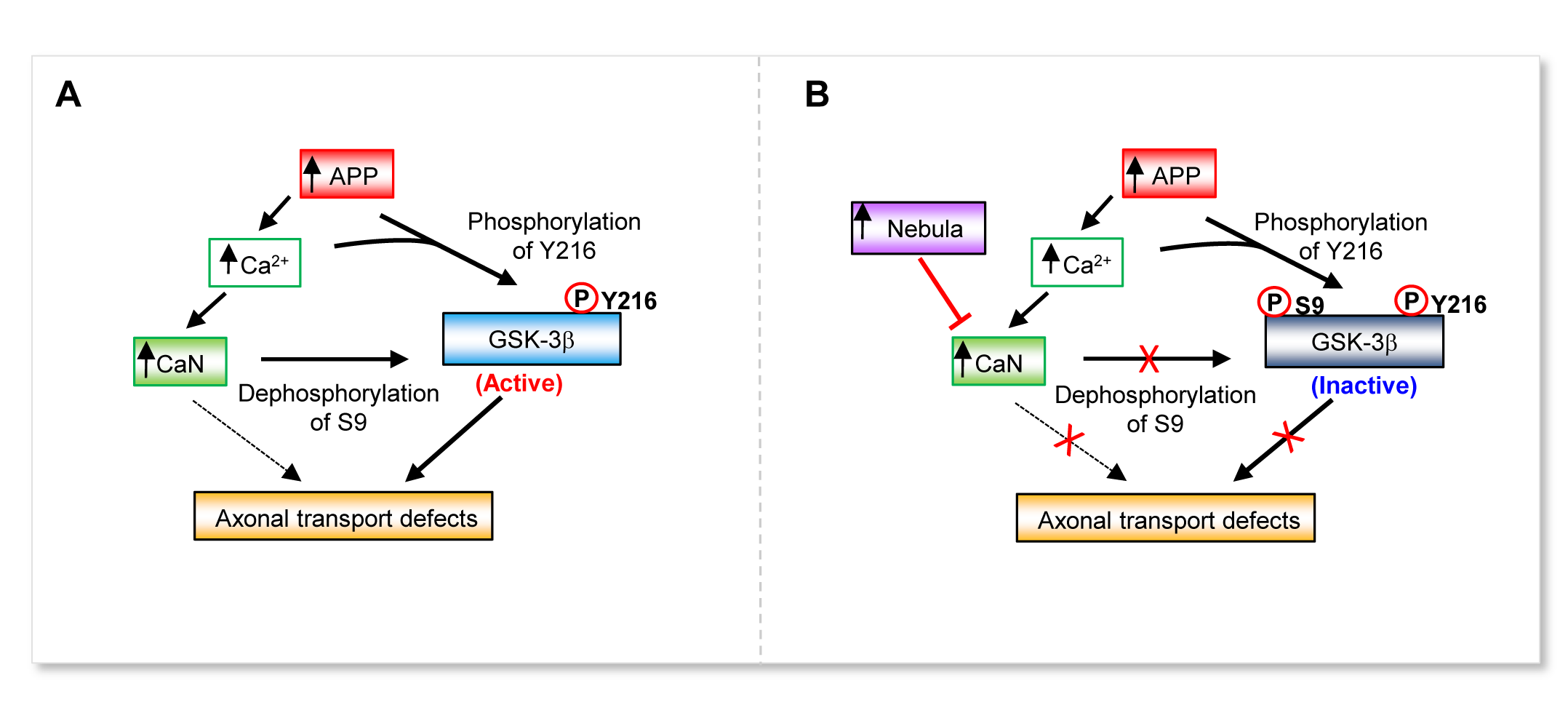

Supplement: Figure S11 — Graphical representation of the interactions between APP, Nebula, calcineurin, and GSK-3β. (A) The left-hand panel depicts downstream signaling events arising from APP upregulation. APP overexpression increases intracellular calcium level which activates calcineurin (CaN) activity and an unknown pathway that leads to phosphorylation of GSK-3β at Y216 (or Y214 in Drosophila). Phosphorylation of GSK-3 atβ Y216 is required for enhancement of GSK-3β activity. Active calcineurin also dephosphorylates GSK-3β at Ser9 to relieve inhibition of GSK-3β, leading to activation of GSK-3β with enhanced activity. GSK-3β activation strongly contributes to axonal transport problems through downstream pathways, whereas calcineurin (independent of GSK-3β pathway) weakly contributes to axonal transport problems. APP upregulation therefore impairs axonal transport through two independent but interacting signaling pathways. (B) The right-hand panel introduces the downstream signaling events arising from interactions between APP and Nebula co-upregulation. Nebula inhibits calcineurin activity, thereby prevents activation of GSK-3β by calcineurin. Even though APP still triggers GSK-3β phosphorylation at Y216, phosphorylation at Ser9 overrides and inhibits activation of GSK-3β. Nebula inhibition of calcineurin rescues APP-mediated axonal transport defects by restoring both GSK-3β and calcineurin activity. (TIF) [file pgen.1003792.s011.tif]
